# District‐level estimation of vaccination coverage: Discrete vs continuous spatial models

**DOI:** 10.1002/sim.8897

**Published:** 2021-02-04

**Authors:** C. Edson Utazi, Kristine Nilsen, Oliver Pannell, Winfred Dotse‐Gborgbortsi, Andrew J. Tatem

**Affiliations:** ^1^ WorldPop, School of Geography and Environmental Science University of Southampton Southampton UK; ^2^ Southampton Statistical Sciences Research Institute University of Southampton Southampton UK

**Keywords:** continuous spatial models, discrete spatial models, district‐level estimation, household surveys, INLA‐SPDE, vaccination coverage

## Abstract

Health and development indicators (HDIs) such as vaccination coverage are regularly measured in many low‐ and middle‐income countries using household surveys, often due to the unreliability or incompleteness of routine data collection systems. Recently, the development of model‐based approaches for producing subnational estimates of HDIs using survey data, particularly cluster‐level data, has been an active area of research. This is mostly driven by the increasing demand for estimates at certain administrative levels, for example, districts, at which many development goals are set and evaluated. In this study, we explore spatial modeling approaches for producing district‐level estimates of vaccination coverage. Specifically, we compare discrete spatial smoothing models which directly model district‐level data with continuous Gaussian process (GP) models that utilize geolocated cluster‐level data. We adopt a fully Bayesian framework, implemented using the INLA and SPDE approaches. We compare the predictive performance of the models by analyzing vaccination coverage using data from two Demographic and Health Surveys (DHS), namely the 2014 Kenya DHS and the 2015‐16 Malawi DHS. We find that the continuous GP models performed well, offering a credible alternative to traditional discrete spatial smoothing models. Our analysis also revealed that accounting for between‐cluster variation in the continuous GP models did not have any real effect on the district‐level estimates. Our results provide guidance to practitioners on the reliability of these model‐based approaches for producing estimates of vaccination coverage and other HDIs.

## 1 INTRODUCTION

Health and development indicators (HDIs) such as vaccination coverage, child mortality, educational attainment and access to resources and basic services^1^, ^2^ are regularly monitored within countries to inform policy and decision‐making and evaluate progress towards key development goals. Since the launch of the Sustainable Development Goals (SDGs) in 2015^1^ with the central goal of “leaving no one behind”, there has been an increasing recognition of the importance of geographical precision in the estimation of HDIs within the global health and development community. Spatially detailed estimates of HDIs are key to understanding the inequities that exist within countries, which are often masked by country level and regional estimates. In many low‐ and middle‐income countries where routine data sources can often be incomplete and unreliable, subnational estimates of HDIs are produced regularly using nationally representative household surveys such as the Demographic and Health Surveys (DHS), Multiple Indicator Cluster Survey (MICS), and Living Standards Measurement Survey (LSMS),^3^, ^4^, ^5^ which are typically conducted every 5 years.

Classical approaches used in estimating HDIs from household survey data include direct weighted estimators, routinely used by the DHS program and other survey programs. Direct survey estimation approaches account for the survey design but the resulting estimates can have large variances, particularly where data are sparse. Most surveys are designed to be representative at the national and first administrative levels, hence estimates of HDIs cannot often be produced at the second administrative level (usually districts), at which data are needed for program planning, implementation, monitoring, and evaluation in many contexts. Small area estimation (SAE) methods such as spatial smoothing models^6^, ^7^, ^8^, ^9^, ^10^, ^11^ acknowledge the survey design but also allow for borrowing strength across neighboring areas to produce more reliable estimates with smaller variances. Other model‐dependent SAE approaches^12^, ^13^ rely on additional data from censuses and other administrative sources. This makes their use more challenging since censuses are only conducted every decade, rarely line up temporally with surveys, and detailed data can be difficult to obtain due to confidentiality constraints. Discrete spatial models are, however, dependent on the neighborhood structure of the areas for which estimates are produced, which can be arbitrary and subject to irregular changes in many settings.^14^ Also, as with direct survey estimates, these models are fitted at spatial scales at which the survey is deemed representative, and estimates can be produced for a given administrative level at a time—usually the most detailed level possible.

Many household surveys are now geolocated and the availability of data on survey cluster locations, though typically displaced spatially for confidentiality reasons, has facilitated the use of continuous spatial models for the production of estimates of HDIs.^15^, ^16^, ^17^, ^18^, ^19^, ^20^, ^21^ These models are fitted using the cluster‐level survey data, and include a continuous Gaussian process (GP) random effect to account for dependence over space. It is a common practice to include a variety of geospatial covariates in these models to boost predictive performance. Estimates of HDIs can be produced at a high resolution (eg, 1 or 5 km), offering the flexibility of aggregation to more operationally relevant administrative units. Moreover, the standardized spatial scales at which estimates can be produced regardless of geography enable integration with other data sets, opening up opportunities for further research and operationalization. However, these models often do not account for the survey design, as the application of sampling weights is feasible only when producing estimates at the area level in most surveys. Nevertheless, it is supposed that some geospatial covariates included in these models may implicitly adjust for some aspects of the survey design such as urban‐rural stratification.^22^

To date, little is known about the comparative performance of these model‐based approaches used for estimating HDIs, particularly how continuous GP models compare with the more traditional spatial smoothing models for producing district‐level estimates, which are crucial for planning and decision making. Very little published work (eg, Paige et al^22^) compare these approaches, and in the context of vaccination coverage estimation, we are not aware of any study examining their performance. In this article, we explore the performance of discrete and continuous spatial models for producing district‐level estimates of vaccination coverage using data from the 2014 Kenya DHS^23^ and 2015‐16 Malawi DHS^24^, both of which were designed to be representative at the district level, and therefore constitute ideal example cases. Further, we investigate modeling issues such as the effects of accounting for sampling weights and covariates, and between‐cluster variation in the case of continuous GP models.

The rest of this article is organized as follows. Cluster‐ and district‐level vaccination coverage data as well as geospatial and DHS covariates analyzed in this study are presented in Section 2. Section 3 describes both the discrete and continuous spatial models investigated. In Section 4, the models are applied to mapping vaccination coverage in Kenya and Malawi. We conclude with a discussion of our findings in Section 6.

## 2 DATA

Data on the coverage of the first dose of measles‐containing vaccine (MCV1) and the third dose of diphtheria‐tetanus‐pertussis‐containing vaccine (DTP3) were obtained from the 2014 Kenya DHS^23^ and 2015‐16 Malawi DHS,^24^ respectively, for children 12 to 23 months of age. The 2014 Kenya DHS utilized a stratified, two‐stage sampling design, which involved the selection of clusters (or enumeration areas) from a national sampling frame in the first stage and households from within the clusters in the second stage, with stratification achieved by separating each of the 47 counties into urban and rural strata. In addition to producing estimates at the national and provincial levels, the survey was designed to produce estimates of some indicators at the county or district level, including vaccination coverage. The 2015‐16 Malawi DHS was based on a similar sampling design and is representative for each of the 28 districts within the country.

**FIGURE 1:**
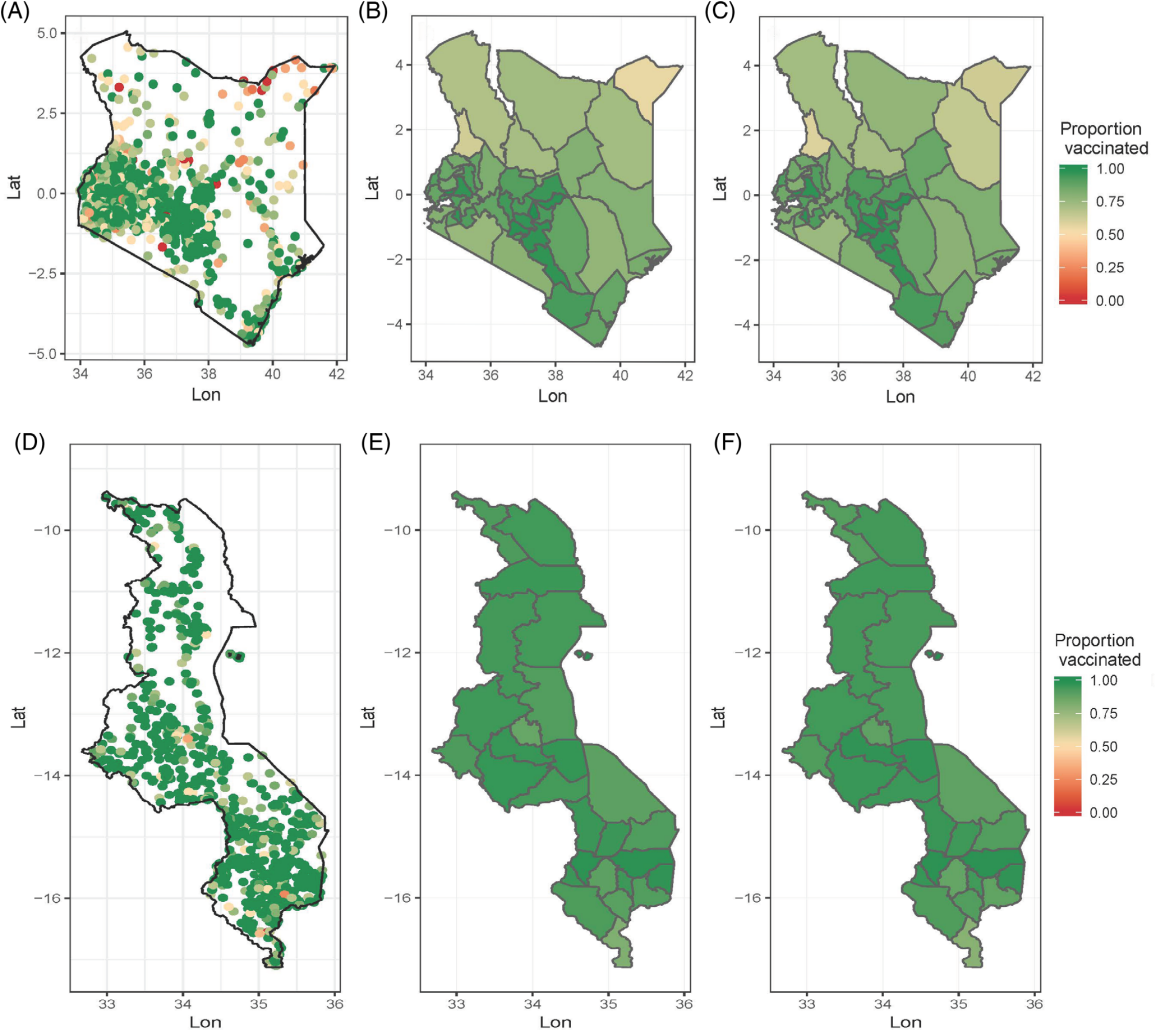
Plots of (A, D) cluster level, (B, E) unweighted district level, and (C, F) direct weighted district‐level estimates of (A‐C) MCV1 and (D‐F) DTP3 coverage for children aged 12 to 23 months using the (A‐C) 2014 Kenya DHS and (D‐F) 2015‐16 Malawi DHS [Color figure can be viewed at wileyonlinelibrary.com]

For each vaccine, we determined the vaccination status of each child included in these surveys based on information obtained from the child's vaccination card or verbal reports provided by the mother or caregiver. Other information obtained include each child's age in months, the survey sampling weights and the geographical coordinates of the clusters from which each child's household was selected. These individual‐level data were then summarized at both the cluster and district levels to produce the data modeled in this work. At the cluster level, we aggregated the individual‐level data to produce numbers of children surveyed, numbers vaccinated and corresponding proportions of children vaccinated (ie, vaccination coverage). As in previous work,^18^, ^19^ we excluded clusters where ≤1 child was surveyed from both surveys. At the district level, we calculated corresponding unweighted and direct weighted estimates (see modeling section). The resulting cluster‐ and district‐level vaccination coverage data are displayed in Figure 1. There are more clusters with lower coverage levels in Kenya compared with Malawi. At the district level, DTP3 coverage in Malawi (Figure 1E and F) is higher and more homogeneous compared with MCV1 coverage in Kenya (Figure 1B and C).

To improve the prediction of vaccination coverage using the approaches investigated, we obtained some geospatial covariate information successfully used in previous work,^23^, ^25^, ^26^ in some cases as proxies for a variety of factors, to model and predict vaccination coverage. These include travel time to the nearest health facility (Kenya), night‐time lights (Kenya and Malawi), slope (Kenya), and poultry density (Kenya)—see supplementary Figure 1 and supplementary Table1. For each country, these covariates were processed and harmonized at 5 × 5 km resolution, at which grid‐level estimates were produced in this work. To extract the values of the covariates for each cluster location, we used the approach described in Utazi et al^18^ and Perez‐Haydrich et al^27^, which accounts for the displacement of the clusters (this displacement often occurs within districts in DHS surveys^28^). Furthermore, owing to the little variation in the spatial distribution of DTP3 coverage in Figures 1D‐F, we obtained additional covariate information known to be associated with coverage^29^, ^30^ from the Malawi DHS, in order to boost predictive performance. These covariates include: proportion of children who owned a vaccination card and/or a health document, proportion of households belonging to the top three wealth quintiles (household wealth) and proportion of mothers who had at least a primary education (mother's education). We first calculated these covariates at the cluster level using detailed definitions provided in supplementary Table 1 and then used the “krig” function in the fields package^31^ in R to create corresponding 5 × 5 km interpolated surfaces, with the optimal range parameter set to the first quartile of the distances between the clusters (other quartiles yielded almost the same results). The interpolation was carried out using logit‐transformed cluster‐level data in each case, after which the estimates were back‐transformed to the unit interval.

At the district level, the values of the geospatial covariates were obtained as population‐weighted averages over all the 5 × 5 km grid points falling within each district, as described in the modeling section. The use of population‐weighted aggregation helps to account for the uneven distribution of populations within the districts, although this may not eliminate the potential bias likely to result from the spatial misalignment between the grids and the districts. However, this is less of a concern here since our interest is in predictive inference. The population data used for this covariate‐processing step and for aggregating the grid‐level predictions to the district and other administrative levels (see modeling section) were obtained from WorldPop^32^ and processed at 5 × 5 km resolution. These were 2014 and 2015 estimates of numbers of children aged under 5 years (U5s) for Kenya and Malawi, respectively, which we used as a proxy for the 12‐ to 23‐month age group. District‐level estimates of the three covariates derived from the Malawi DHS were obtained as direct survey estimates, accounting for the complex survey design—see modeling section. Plots of all the covariates are shown in supplementary Figures 1 and 2 at 5 × 5 km resolution. In our analyses, we log‐transformed some of the covariates to encourage normality and improve their relationships with vaccination coverage (see supplementary Tables 2 and 4).

## 3 MODEL SPECIFICATION

Here, we describe the spatial models investigated in this work. We first describe the discrete spatial models (for the district‐level data) that assume a fixed, discrete spatial domain and model spatial dependence using an areal random effect. Next, we present the continuous spatial models (for the point or cluster‐level data) that assume a fixed, continuous spatial domain and account for spatial dependence using a continuous GP random effect.

### 3.1 Discrete spatial models

Let $A_{1},\ldots ,A_{n_{A}}$ denote the $n_{A}$ districts of the study region $A\subset {\mathbb{R}}^{2}$. The first model that we investigate is a naïve approach, which does not account for the survey design. It assumes that the areal observations, that is, the numbers of children vaccinated within each of the districts, denoted by $Y\left(A_{1}\right),\ldots ,Y\left(A_{n_{A}}\right)$, follow a binomial spatial regression model given by

$$Y\left(A_{i}\right)∼\text{Binomial}\left(N\left(A_{i}\right),,,p\left(A_{i}\right)\right),i=1,\ldots ,n_{A},$$

(1) $$\text{logit}\left(p\left(A_{i}\right)\right)=x^{\prime }\left(A_{i}\right)\beta +\varphi \left(A_{i}\right),$$

where $N\left(A_{1}\right),\ldots ,N\left(A_{n_{A}}\right)$ and $p\left(A_{1}\right),\ldots ,p\left(A_{n_{A}}\right)$ are the corresponding numbers of children surveyed and the underlying probabilities of being vaccinated (ie, vaccination coverage) within the districts, and $x^{\prime }\left(A_{i}\right)$ is a *p* ‐dimensional covariate vector associated with area $A_{i}$. The spatial random effects $\varphi =\left(\varphi \left(A_{1}\right),\ldots ,\varphi \left(A_{n_{A}}\right)\right)$ are included in the model to account for residual spatial autocorrelation. Throughout, these are assigned a conditional autoregressive (CAR) prior proposed by Leroux et al,^33^ which was shown to outperform other popular choices such as the BYM model in disease mapping studies.^34^ The Leroux model is given by $\varphi ∼N\left(0,\;\sigma_{\varphi }^{2}Q^{-1}\left(W\right)\right)$, where $Q{\left(.\right)}_{n_{A}\times n_{A}}$ is the precision matrix and $\sigma_{\varphi }^{2}$ is a variance parameter. More explicitly, $Q\left(W\right)=\rho \left(\mathrm{diag}\left(W1\right)-W\right)+\left(1-\rho \right)I_{n_{A}}$, where *ρ* is a spatial autocorrelation parameter, **1** is an $n_{A}$ vector of 1's, $I_{n_{A}}$ is an identity matrix and ***W*** is a binary matrix characterizing the neighborhood structure of the districts. That is, *W*_*ij*_ = 1 if areas $A_{i}$ and $A_{j}$ share a common border and zero otherwise. Typically, 0 ≤ *ρ* ≤ 1, with *ρ* = 0 corresponding to an independent and identically distributed (iid) model and *ρ* = 1 corresponding to strong spatial dependence, also known as an improper intrinsic model.^34^ We also refer to discrete spatial model (1) as an unweighted binomial (D‐UNWB) model.

The second model that we consider accounts for the survey design by modeling the empirical logistic transform of the direct survey estimator ${\hat{p}}_{\mathrm{DIR}}\left(A_{i}\right)$, $Y^{L}\left(A_{i}\right)=\log\left[{\hat{p}}_{\mathrm{DIR}}\left(A_{i}\right)/\left(1-{\hat{p}}_{\mathrm{DIR}}\left(A_{i}\right)\right)\right]$ as follows

$$Y^{L}\left(A_{i}\right)|\eta \left(A_{i}\right)∼N\left(\eta \left(A_{i}\right),,,{\hat{\sigma }}_{Y}^{2}\left(A_{i}\right)\right),$$

(2) $$\eta \left(A_{i}\right)=x^{\prime }\left(A_{i}\right)\beta +\varphi \left(A_{i}\right),$$

where ${\hat{\sigma }}_{Y}^{2}$ is the known (design‐based) variance associated with $Y^{L}\left(A_{i}\right)$. That is, ${\hat{\sigma }}_{Y}^{2}\left(A_{i}\right)={\hat{\sigma }}_{p_{\mathrm{DIR}}}^{2}\left(A_{i}\right)/{\left[{\hat{p}}_{\mathrm{DIR}}\left(A_{i}\right)\left(1-{\hat{p}}_{\mathrm{DIR}}\left(A_{i}\right)\right)\right]}^{2}$, where the Horvitz‐Thompson estimator ${\hat{p}}_{\mathrm{DIR}}\left(A_{i}\right)=\sum_{k\in A_{i}}{\tilde{w}}_{\mathrm{ki}}Y_{\mathrm{ki}}/\sum_{k\in A_{i}}{\tilde{w}}_{\mathrm{ki}}$ ^35^ is calculated using the normalized sampling weights ${\tilde{w}}_{\mathrm{ki}}$ (the sampling weight is the inverse probability of inclusion in the sample adjusted for (unit) nonresponse^36^) and binary outcomes (vaccinated or not) *Y*_*ki*_ for each *k* individual in area $A_{i}$. Furthermore, ${\hat{\sigma }}_{p_{\mathrm{DIR}}}^{2}\left(A_{i}\right)$ is the variance of ${\hat{p}}_{\mathrm{DIR}}\left(A_{i}\right)$, which usually has a complex form under complex surveys and is estimated by linearization or the use of replication weights.^37^ Throughout, the sampling weights are normalized to sum up to the sample size in each area, that is, . $\sum_{k\in A_{i}}{\tilde{w}}_{\mathrm{ki}}=N\left(A_{i}\right)$, as is the case in previous studies.^6^, ^9^, ^10^ In model (2), which is also known as the logit‐normal (D‐LN) model,^8^, ^9^, ^10^ the quantity of interest is the posterior distribution of the district‐level vaccination coverage given by $p_{\eta }\left(A_{i}\right)=\exp\left(\eta \left(A_{i}\right)\right)/\left(1+\exp\left(\eta \left(A_{i}\right)\right)\right)$. The model is known to mitigate some of the high variance problems often associated with the direct estimates, ${\hat{p}}_{\mathrm{DIR}}\left(.\right)$.^22^ However, its limitation is that boundary estimates (these occur when ${\hat{p}}_{\mathrm{DIR}}\left(A_{i}\right)=0/1)$ yield undefined variances^22^ and these introduce missing values in the data that are modeled. Although ad hoc measures, such as augmenting the numerator with an additional sampled individual possessing the attribute of interest when ${\hat{p}}_{\mathrm{DIR}}\left(A_{i}\right)=0$ as suggested in Mercer et al,^8^ could be used to deal with this problem, we have chosen here to treat those cases as missing observations which are estimated during model‐fitting. In our examples, this problem occurred only in one district in Kenya and is, hence, unlikely to have any real effect on both parameter estimation and the modeled estimates.

The third model is based on the binomial sampling distribution as in model (1) but uses the “effective sample size” to account for the sampling design. Chen et al^6^ obtained the effective sample size as the sample size that is required to make the variance under a complex design equivalent to that of a simple random sample. The effective sample size model (D‐ESS) can be expressed as

$$Y^{E}\left(A_{i}\right)∼\text{Binomial}\;\left(N^{E}\left(A_{i}\right),,,\tilde{p}\left(A_{i}\right)\right),$$

(3) $$\text{logit}\left(\tilde{p}\left(A_{i}\right)\right)=x^{\prime }\left(A_{i}\right)\beta +\varphi \left(A_{i}\right),$$

where $N^{E}\left(A_{i}\right)=$ ${\hat{p}}_{\mathrm{DIR}}\left(A_{i}\right)\left(1-{\hat{p}}_{\mathrm{DIR}}\left(A_{i}\right)\right)/{\hat{\sigma }}_{p_{\mathrm{DIR}}}^{2}\left(A_{i}\right)$ is the effective sample size for area $A_{i}$, and $Y^{E}\left(A_{i}\right)=N^{E}\left(A_{i}\right)\times {\hat{p}}_{\mathrm{DIR}}\left(A_{i}\right)$ is the effective number of cases. By using $N^{E}\left(A_{i}\right)$ and $Y^{E}\left(A_{i}\right)$ instead of the actual values as in (1), the model acknowledges the variable information that each child contributes under a complex survey design. We note that similar to model (2), model (3) is also affected by boundary problems, which we dealt with as in model (2); see Chen et al^6^ for other solutions to the problem.

Additional information on the models described here can be found elsewhere.^6^, ^8^, ^10^ Further, to address the spatial misalignment between the geospatial covariates (see Section 2) and the districts in models (1), (2), (3)), we produced the district‐level covariate values $x_{r}\left(A_{i}\right);r=1,\ldots ,p-1$, as population‐weighted averages over all the grid cells whose centroids fall within each district. That is, $x_{r}\left(A_{i}\right)=\int_{A_{i}}x_{r}\left(s\right)\times q\left(s\right)ds$, which is approximated using numerical integration as $x_{r}\left(A_{i}\right)\approx \sum_{j=1}^{m_{i}}x_{r}\left(s_{j}\right)\times q\left(s_{j}\right)$, where *m*_*i*_ is the number of grid locations with centroids in district $A_{i}$ and *q*(***s***) is the proportion of the population of district $A_{i}$ at grid location ***s*** . We note that if the *j* th grid cell lies at the boundary of the district, *q*(***s***_*j*_) could also be possibly obtained as the proportion of the district population living within the area of intersection.

### 3.2 Continuous spatial models

The geostatistical approach to producing estimates of survey indicators utilizes the same methodology described in previous work.^19^ Letting $s_{1},\ldots ,s_{n_{c}}\;\left(s\in A\subset {\mathbb{R}}^{2}\right)$ denote the survey cluster locations (ie, the longitude‐latitude coordinates), the geostatistical model can be expressed as

$$Y\left(s_{i}\right)∼\text{Binomial}\left(N\left(s_{i}\right),,,p\left(s_{i}\right)\right),$$

(4) $$\text{logit}\left(p\left(s_{i}\right)\right)=x{\left(s_{i}\right)}^{T}\beta +\omega \left(s_{i}\right)+\epsilon \left(s_{i}\right),$$

where *p*(***s***_*i*_) denotes vaccination coverage at location ***s***_*i*_ , *ϵ*(***s***_*i*_) is an iid Gaussian random effect (or a nugget term) with variance, $\sigma_{\epsilon }^{2}$, used to model nonspatial residual variation (or between‐cluster or excess binomial variation), *ω*(***s***_*i*_) is a continuous Gaussian process spatial random effect used to capture residual spatial correlation in the model, that is, $\omega ={\left(\omega \left(s_{1}\right),\ldots ,\omega \left(s_{n_{c}}\right)\;\right)}^{T}∼\mathrm{GP}\left(0,\sum_{\omega }\right)$, and other variables are as defined previously. Further, ∑_*ω*_ is assumed to follow the Matérn covariance function^25^ given by $\sum_{\omega }\left(s_{i},s_{j}\right)=\frac{\sigma_{\omega }^{2}}{2^{\nu -1}\Gamma \left(\nu \right)}{\left(\kappa \|s_{i}-s_{j}\|\right)}^{\nu }\;K_{\nu }\;\left(\kappa \|s_{i}-s_{j}\|\right)$, where ‖.‖ denotes the Euclidean distance between cluster locations ***s***_*i*_ and $s_{j},\;\sigma_{\omega }^{2}>0$ is the marginal variance of the spatial process, *κ* is a scaling parameter related to the range $r\left(r=\frac{\sqrt{8\nu }}{\kappa }\right)$—the distance at which spatial correlation is close to 0.1, and *K*_*ν*_ is the modified Bessel function of the second kind and order *ν* > 0. We set *ν* = 1 for identifiability reasons, as is often the case.^38^ We note that geostatistical modeling using cluster‐level household survey data such as the DHS do not usually incorporate the sampling weights. This is mostly because sampling weights are made available at the cluster level, which are usually the same for all individuals within the same cluster.

Finally, the last model that we consider is a variant of model (4) which does not include the iid term *ϵ*(***s***_*i*_) used to account for excess binomial variation. Its linear predictor is given by

(5) $$\text{logit}\left(p\left(s_{i}\right)\right)=x{\left(s_{i}\right)}^{T}\beta +\omega \left(s_{i}\right).$$

Model (5) is included in this study to investigate the effect of accounting for between‐cluster variation on the district‐level estimates. Subsequently, we also refer to continuous spatial models (4) and (5) as the C‐GPIID and C‐GP models, respectively.

Predictions using models (4) and (5) were first produced at 5 × 5 km resolution as mentioned previously. District‐level predictions using these models were obtained as population‐weighted averages taken over all the grid cells falling within each district. That is, for $i=1,\ldots ,n_{A}$, $p^{r}\left(A_{i}\right)=\int_{A_{i}}p^{r}\left(s\right)\times q\left(s\right)ds\approx \sum_{j=1}^{m_{i}}p^{r}\left(s_{j}\right)\times q\left(s_{j}\right)$, where *m*_*i*_ and *q*(***s***) are as defined previously, and *r* denotes the *r*th posterior sample (see supplementary materials for details).

## 4 BAYESIAN INFERENCE, COMPUTATION, AND MODEL EVALUATION

Models (1), (2), (3), (4), (5)) were implemented in a Bayesian framework. We assigned the following priors to the parameters: ***β*** ∼ *N*(**0**, 10^6^ ***I***); log(*ρ*/(1 − *ρ*)) ∼ *N*(0, 0.45); $\log\left(\sigma_{\varphi }^{-2}\right)∼\text{logGamma}\left(0.1,0.1\right)$. We placed a penalized complexity (PC) prior introduced in Simpson et al^39^ on *σ*_*ϵ*_ such that *p*(*σ*_*ϵ*_ > 5) = 0.01. Similarly, following Fuglstad et al^40^, a joint PC prior was placed on the covariance parameters of the spatial random effect, ***ω*** . These were: *p*(*r* < *r*_0_) = 0.01 and *p*(*σ*_*ω*_ > 5) = 0.01, with *r*_0_ chosen to be 5% of the extent of each of Kenya and Malawi in the north‐south direction, with the aim of capturing a moderate level of spatial dependence in the model. In both applications, the district‐level estimates were found to have negligible sensitivity to the prior on $\sigma_{\varphi }^{-2}$.

The models were fitted using the INLA and SPDE^38^, ^41^ approaches implemented in the R‐INLA package. INLA is a faster alternative to the traditional MCMC for performing approximate Bayesian inference. The INLA approach produces a numerical approximation of the marginal posterior distributions of each of the unknown quantities in the models. The SPDE approach is particularly required for the estimation of the Gaussian spatial random effect, ***ω*** . Further details of the implementation of the INLA‐SPDE approach are provided in supplementary materials. We note that in R‐INLA, any missing values in the data are automatically estimated during model‐fitting using their posterior predictive distributions; see Gómez‐Rubio,^42^ chapter 12.

To assess the performance of the fitted models for out‐of‐sample prediction, we carried out a leave‐one‐district‐out cross‐validation. For models (4) and (5), this entailed excluding all cluster locations within a given district each time during model‐fitting. Using the observed ( *p* ) and predicted ($\hat{p}$) district‐level vaccination coverage, we computed the following model evaluation metrics: relative bias ($\text{RBias}=\frac{1}{n_{A}}\sum_{i=1}^{n_{A}}\left({\hat{p}}_{i}-p_{i}\right)/p_{i}$, root mean square error ($\text{RMSE}=\sqrt{\sum_{i}{\left({\hat{p}}_{i}-p_{i}\right)}^{2}/n_{A}}$), mean absolute error $\left(\mathrm{MAE}=\frac{1}{n_{A}}\sum_{i=1}^{n_{A}}|{\hat{p}}_{i}-p_{i}|\right)$ and the continuous ranked probability score ($\text{CRPS}\left(F_{i},p_{i}\right)=E_{F_{i}}\left|X_{i}-p_{i}\right|-\frac{1}{2}E_{F_{i}}|X_{i}-X_{i}^{*}|)$,^43^ where *F*_*i*_(.) is the cumulative distribution function corresponding to the predictive distribution of the *i* th district‐level estimate, and *X*_*i*_ and $X_{i}^{*}$ are two independent random variables distributed according to *F*_*i*_(.). With *m* posterior samples, the measure can be estimated as $\text{CRPS}\left(F_{i},p_{i}\right)=\frac{1}{m}\sum_{j=1}^{m}|{\hat{p}}_{i}^{\left(j\right)}-p_{i}|-\frac{1}{2m^{2}}\sum_{j=1}^{m}\sum_{k=1}^{m}|{\hat{p}}_{i}^{\left(j\right)}-{\hat{p}}_{i}^{\left(k\right)}|$, which is then averaged over all the $n_{A}$ districts in each of our applications. We note that the CRPS accounts for both predictive accuracy and uncertainty as it utilizes the whole posterior predictive distribution to measure the discrepancy between the observations and the predictions, unlike the other measures which use a particular summary ${\hat{p}}_{i}$ (eg, the posterior mean) of the predictions. For all the models, we take *p*_(.)_ to be the direct survey estimates ${\hat{p}}_{\mathrm{DIR}}\left(A_{i}\right)$. The closer the values of RBias, MAE, and RMSE are to zero, the better the predictions. In addition, smaller values of CRPS indicate better predictive ability.

R scripts for calculating the input data (using the survey package^44^ for models (2) and (3)) and for model fitting using R‐INLA are provided.

## 5 MAPPING MCV1 AND DTP3 COVERAGE

We now apply models (1), (2), (3), (4), (5)) to produce district‐level estimates of MCV1 and DTP3 coverage for Kenya and Malawi, respectively.

For MCV1/Kenya, across all five models, the parameter estimates presented in supplementary Table 2 show that travel time to the nearest health facility had a significant negative relationship with vaccination coverage. All other covariates were not significant both in the discrete and continuous spatial models. Estimates of the marginal variance of the random effect ***φ*** and the autocorrelation parameter *ρ* were significant in the discrete models (ie, models D‐UNWB, D‐LN, and D‐ESS), with the latter ranging between 0.63 and 0.75, suggesting a strong amount of residual spatial autocorrelation in the fitted models. For the continuous GP models, the estimated spatial range was ≈248 km for the C‐GPIID model and ≈227 km for the C‐GP model (the maximum distance between the clusters is ≈1127 km). This indicates that accounting for between‐cluster variation more likely results in a smoother prediction surface at the grid level. However, the marginal spatial variances for both models are very close, and this parameter is shown to be greater than the iid variance in the C‐GPIID model. Similar patterns were observed when covariates were excluded from the analysis, as reported in supplementary Table 3.

For DTP3/Malawi, the parameter estimates presented in supplementary Table 4 show that none of the covariates were significant in the discrete models. However, in the continuous models, ownership of a health card and/or document and mother's education have significant positive relationships with coverage, consistent with expert knowledge. These differences between the discrete and continuous models in how the covariates relate with coverage, unlike the consistent relationships estimated for Kenya for both classes of models, are likely due to the little variation in DTP3 coverage and/or the effect of spatial misalignment. The estimates of the autocorrelation parameter *ρ* range between 0.51 and 0.53, suggesting a moderate amount of spatial autocorrelation in the discrete models. The estimated spatial ranges for the continuous models are ≈71 and ≈64 km for the C‐GPIID and C‐GP models, respectively, (maximum distance between the clusters is ≈881 km), which indicate the presence of localized spatial dependence in the data and a tendency for the C‐GPIID model to produce slightly smoother modeled estimates. As before, the spatial term is more dominant than the iid term in model C‐GPIID and its marginal variance is somewhat close to that of model C‐GP. However, when covariates were excluded from the analysis, the results in supplementary Table 5 show that the iid term is more dominant in model C‐GPIID, which implies that the covariates mostly accounted for random variation in the model.

Model evaluation statistics reported in Table 1 (based on leave‐one‐district‐out cross‐validation) demonstrate that, for MCV1/Kenya, including covariates in both the discrete and continuous spatial models appears to improve predictive performance slightly, except for model D‐LN and the positive bias in the continuous models. This may have been due to the additional smoothing occasioned by the geospatial covariates at the grid level in the continuous models. For DTP3/Malawi, there is no real evidence of improvement in predictive performance due to the inclusion of covariates in both classes of models, which is most likely due to the little variation in the data. Rather, as for MCV1/Kenya, there is also evidence of positive bias in the continuous models when covariates are included in the analysis. Consequently, all model‐based estimates presented here for DTP3/Malawi are based on the analysis excluding covariates. However, for MCV1/Kenya, these are from the analysis including covariates.

**TABLE 1:** District‐level model validation statistics based on leave‐one‐district‐out cross‐validation

	Covariates included				Covariates excluded			
Model	RMSE	RBias	MAE	CRPS	RMSE	RBias	MAE	CRPS
MCV1/Kenya								
D‐UNWB	0.07	0.002	0.06	0.040	0.07	0.005	0.06	0.041
D‐LN	0.03	−0.009	0.02	0.018	0.03	−0.008	0.02	0.016
D‐ESS	0.07	0.002	0.06	0.041	0.08	0.005	0.06	0.044
C‐GPIID	0.06	0.017	0.05	0.039	0.07	−0.001	0.06	0.043
C‐GP	0.06	0.017	0.05	0.039	0.07	−0.001	0.06	0.044
DTP3/Malawi								
D‐UNWB	0.05	−0.001	0.04	0.025	0.04	0.003	0.03	0.023
D‐LN	0.02	−0.003	0.02	0.011	0.02	−0.004	0.02	0.012
D‐ESS	0.05	−0.001	0.04	0.026	0.04	0.003	0.03	0.023
C‐GPIID	0.05	0.023	0.03	0.027	0.04	−0.002	0.03	0.026
C‐GP	0.05	0.023	0.03	0.027	0.04	−0.004	0.03	0.026

With regard to selecting the best model for both countries/vaccines, there is a perfect agreement among the RMSE, MAE, and CRPS measures, all of which show that the D‐LN model outperformed all the other models both when covariates were included and when these were excluded from the analyses. Interestingly, all three metrics also reveal that the continuous models have very similar predictive performance to models D‐ESS and D‐UNWB for both countries/vaccines, despite utilizing cluster‐level data. Both model D‐UNWB and the continuous models were fitted using unweighted data, suggesting that when using the binomial likelihood, the effect of accounting for sampling weights is negligible. Also, based on all four criteria, the predictive performances of both continuous GP models, that is, models C‐GPIID and C‐GP are almost indistinguishable for both countries/vaccines. This implies that the effect of accounting for between‐cluster variation is negligible when producing aggregate estimates of vaccination coverage, particularly at the district level investigated here. For in‐sample prediction, the plots in supplementary Figures 3 and 4 show good correspondences between the direct estimates and modeled estimates for all the models and both countries/vaccines. In general, apart from the D‐LN model which clearly outperformed all other models, the continuous GP spatial models produced results that are comparable with those of other discrete spatial models investigated.

**FIGURE 2:**
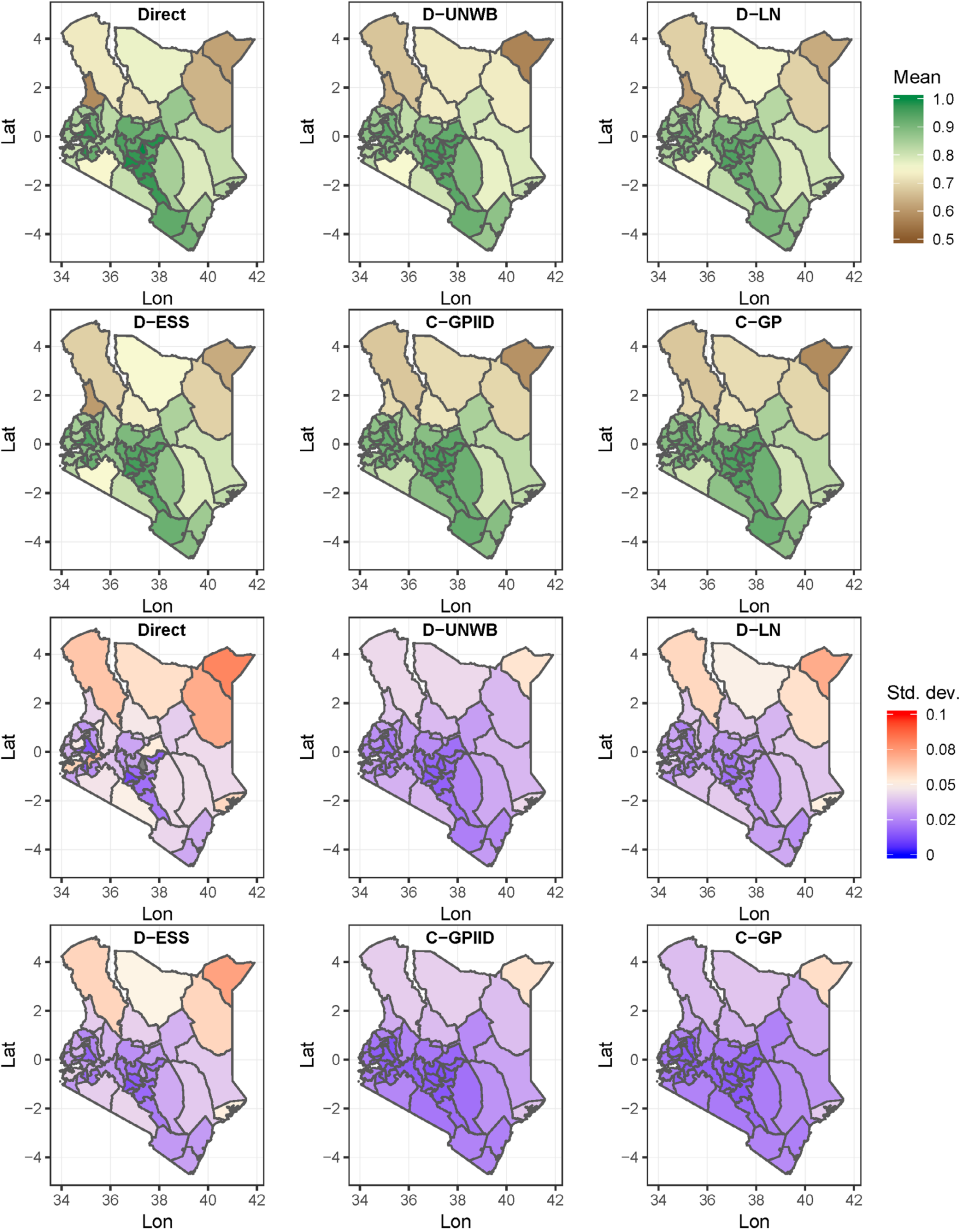
Maps of district‐level estimates of MCV1 coverage and corresponding uncertainties (ie, standard deviations) for Kenya produced using different approaches. Missing data are colored in gray in the uncertainty maps [Color figure can be viewed at wileyonlinelibrary.com]

**FIGURE 3:**
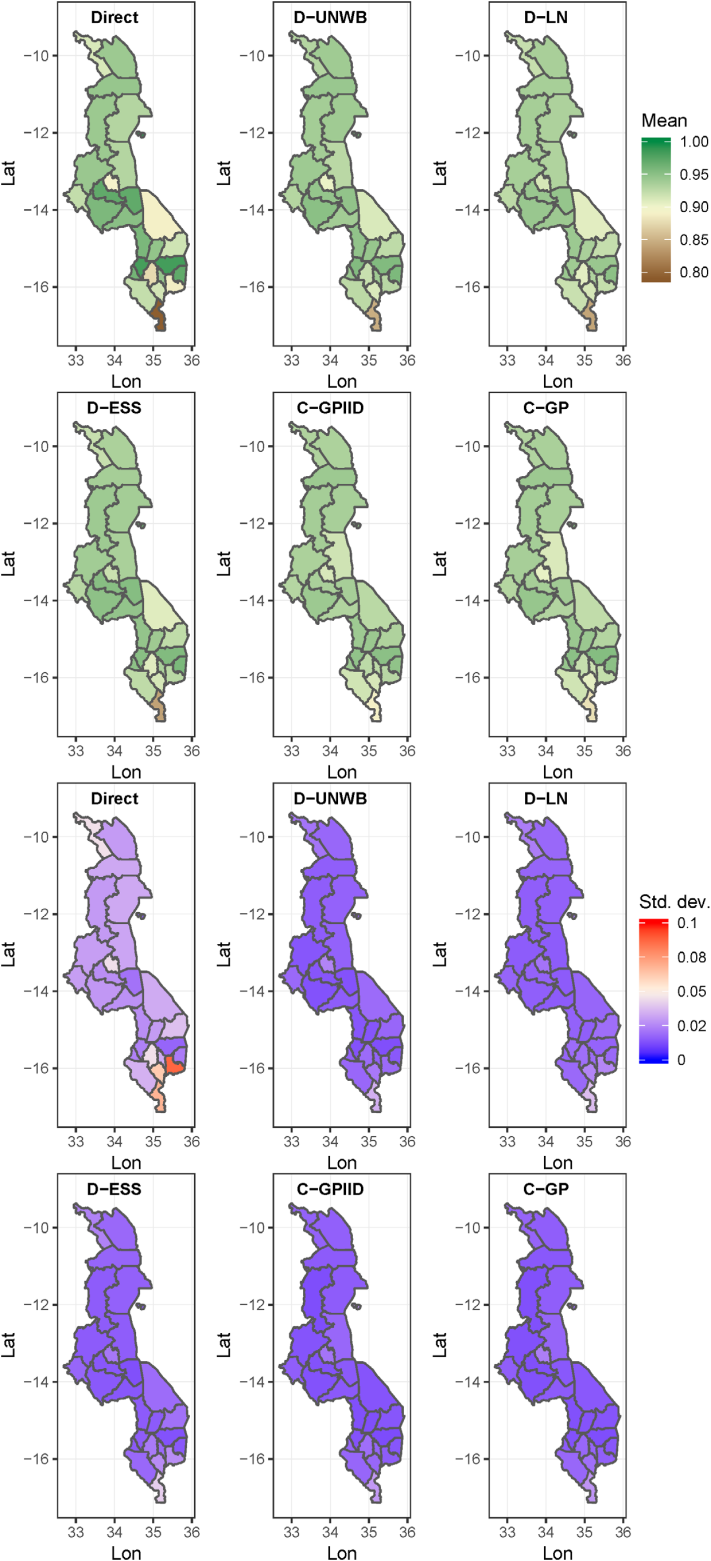
Maps of district‐level estimates of DTP3 coverage and corresponding uncertainties (ie, standard deviations) for Malawi produced using different approaches [Color figure can be viewed at wileyonlinelibrary.com]

District‐level estimates of MCV1 and DTP3 coverage (ie, the posterior means) and associated uncertainties produced using all five models as well as the direct estimates are mapped in Figures 2 and 3, respectively. For MCV1/Kenya, these maps reveal higher coverage in much of southern Kenya, particularly districts within and around the central province, compared with the north. This north‐south gradient in coverage appears to be better elucidated by the continuous GP models. The corresponding standard deviation maps show that the estimates have low uncertainty (≤10% error) as expected, since the survey was designed to be representative at this level. However, it is also clear that the continuous GP models and model D‐UNWB have much lower uncertainties on average, suggesting that weighting, though important in the discrete models to ensure the representativeness of the data, likely increases prediction error. In general, the estimates produced using the different model‐based approaches are broadly similar to the direct estimates, although some differences are apparent due to smoothing (see supplementary Figures 3 and 5).

For DTP3/Malawi, there exists little variation in coverage, although Figure 3 shows that the southernmost district, Nsanje, consistently has the lowest coverage across all the approaches. In general, the modeled estimates are all smoother than the direct estimates and these are broadly similar across all five models (see supplementary Figures 4 and 6). The standard deviations of the estimates are small (<2% in most cases) and the patterns in these are similar for all the models due to the overall high coverage levels (binomial models tend to yield smaller uncertainties for estimates closer to the endpoints of the unit scale). However, the model‐based estimates have lower uncertainties compared with the direct estimates, which demonstrates the utility of the models for producing more precise estimates, as noted previously.

In supplementary Figures 7 and 8, the 5 × 5 km estimates produced using the C‐GPIID model are compared with the corresponding aggregated district‐ and provincial‐level estimates for both countries/vaccines. The figures show how significant heterogeneities in coverage are lost when estimates are produced at coarser spatial scales, particularly for Kenya/MCV1 coverage. Nevertheless, for MCV1, Figure 7 also shows that at the grid level, estimates are more uncertain in areas where data are sparse and the precision of the estimates produced increases dramatically with decreasing geographical precision. Similar patterns can be seen in Malawi/DTP3 coverage in Figure 8, but the estimates are more precise at the grid level, possibly due to the higher coverage levels for this country.

**FIGURE 4:**
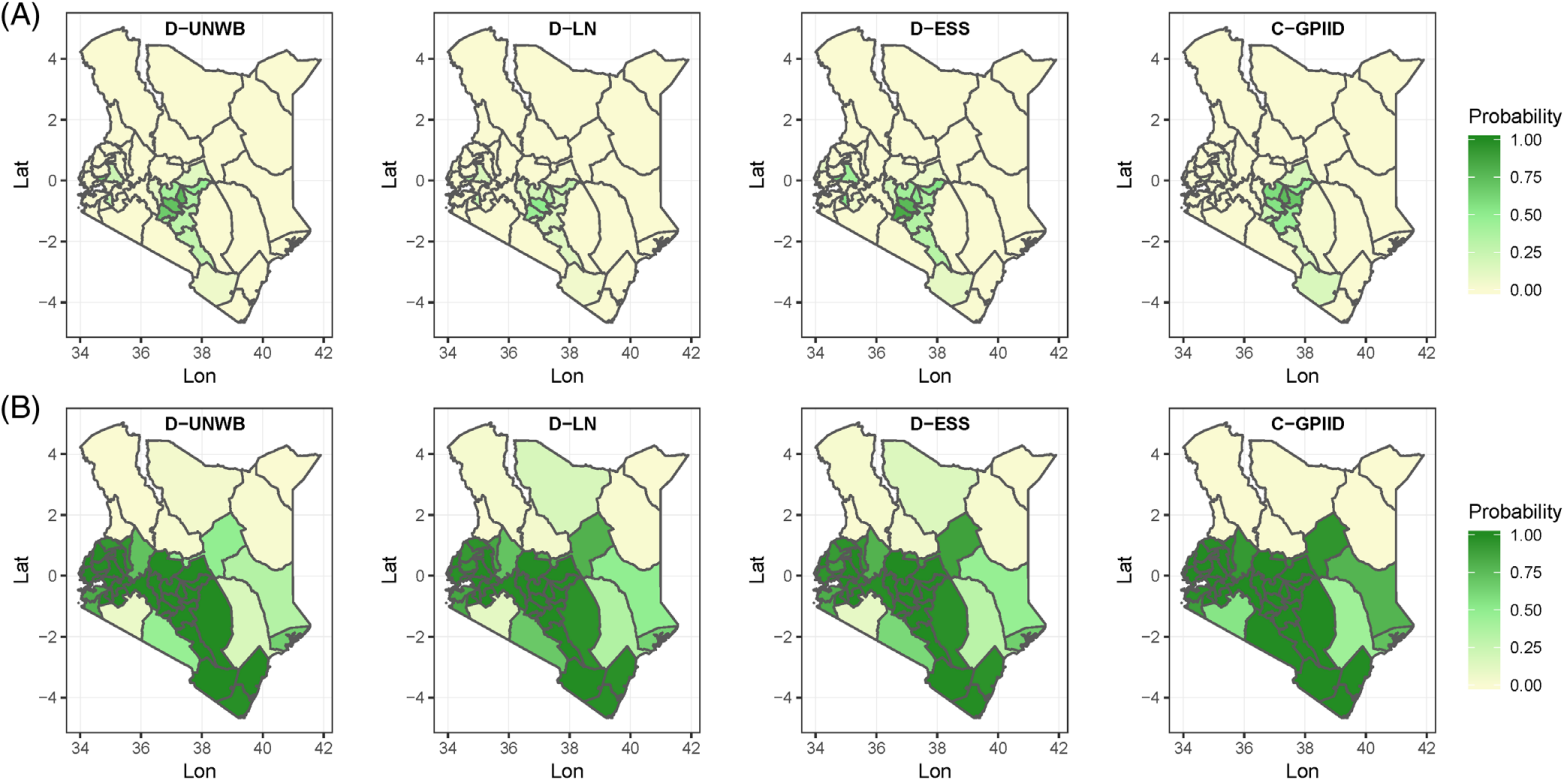
(A) Probability of attaining 95% coverage and (B) probability of attaining 80% coverage for MCV1/Kenya [Color figure can be viewed at wileyonlinelibrary.com]

**FIGURE 5:**
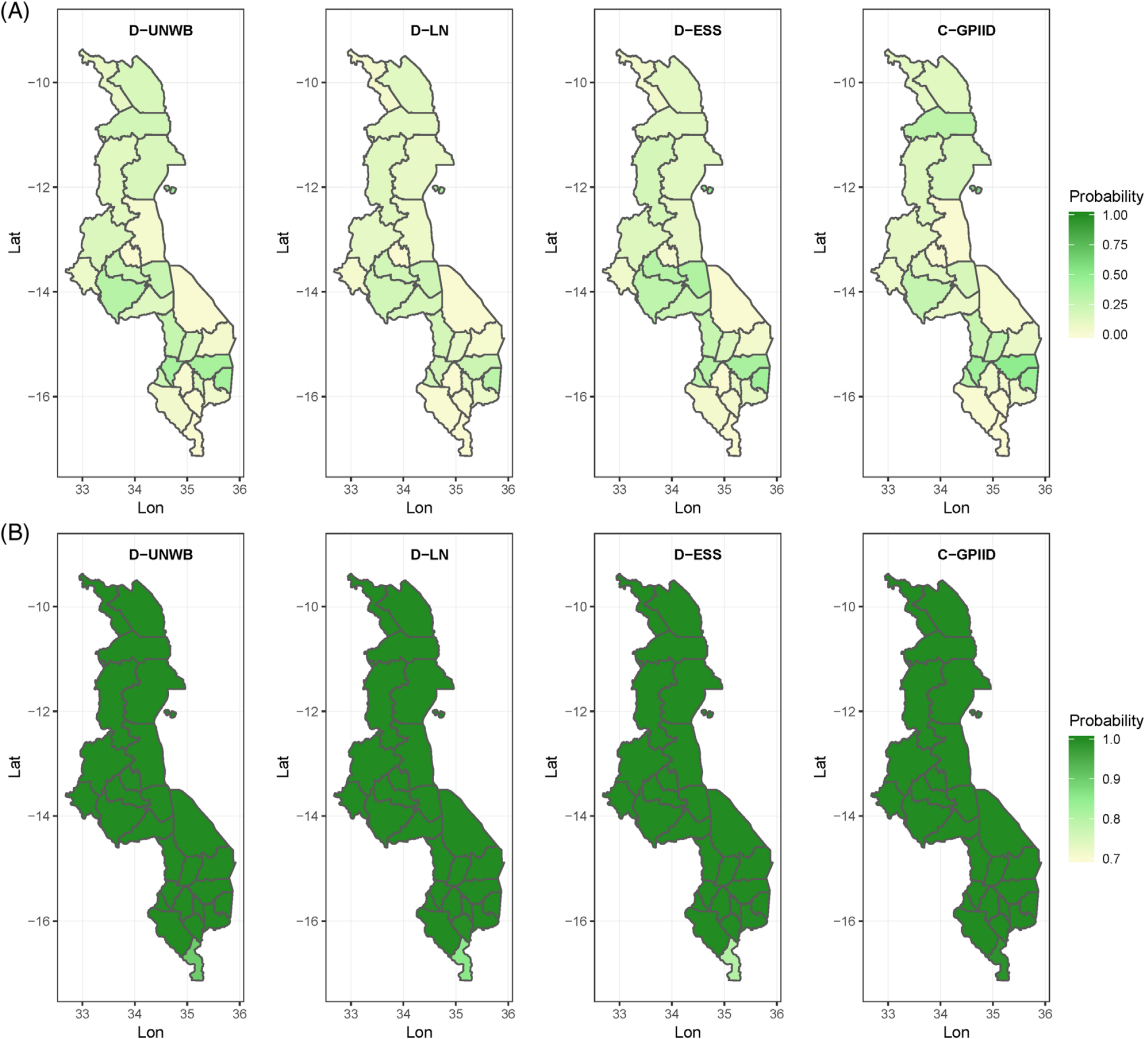
(A) Probability of attaining 95% coverage and (B) probability of attaining 80% coverage for DTP3/Malawi [Color figure can be viewed at wileyonlinelibrary.com]

Understanding districts that have achieved certain targets is often of great interest in the estimation of vaccination coverage and other HDIs,^2^, ^19^ especially for monitoring progress towards the SDGs. In Figures 4 and 5, we map the probabilities of attaining different coverage thresholds for each district in both countries using four of the models investigated. These maps are an alternative way to visualize the uncertainties associated with the coverage estimates whilst at the same time answering important programmatic questions. When using the 95% coverage target, it can be seen that for MCV1/Kenya, districts with a high probability of attaining the threshold are concentrated mostly in the Eastern and Central provinces and these are similar across the four models, although model D‐LN estimates slightly lower probabilities for those districts. With the 80% threshold, the north‐south divide is again apparent and this is more pronounced with models D‐UNWB and C‐GPIID. For DTP3/Malawi, there is a strong evidence that nearly all the districts had attained 80% coverage across all four models in Figure 5B. Further, the probabilities of reaching the 95% coverage threshold mapped in Figure 5A are very similar across all the models and these are shown to be rather low for many districts. In general, the patterns seen in both figures are broadly similar across both classes of models investigated. We note that this probabilistic interpretation of the coverage estimates is a desirable advantage that the Bayesian model‐based framework offers over the direct estimates.

## 6 DISCUSSION

The production of spatially resolved estimates of HDIs has become an active area of research in recent times, not least because of the SDGs' leave no one behind agenda, which has led to an increase in demand for such estimates for various purposes, including monitoring and evaluating progress towards these goals. Amid this development, district‐level estimates of HDIs have gained a vital role since many development goals are often set and monitored at this administrative level. For example, the new Immunization Agenda 2030^45^ clearly recognizes the importance of achieving high equitable coverage at the district level. In this work, we have evaluated the performance of discrete and continuous spatial models for the production of district‐level estimates of vaccination coverage. We checked for differences in the district‐level estimates produced using the different models and compared the modeled estimates with the direct survey estimates, which are usually considered to be the gold standard.^22^

Our analyses revealed that the continuous GP models which utilized cluster‐level data performed well in comparison with the discrete spatial models which were fitted using district‐level data. This shows that despite the ubiquity of direct estimates and discrete spatial smoothing models used in SAE, continuous GP models are a credible alternative for producing district‐level estimates of HDIs using household survey data. This is especially so in settings where surveys are not representative at the district level, which precludes the use of discrete models. The continuous GP modeling framework offers the additional advantage of producing estimates in a standardized format irrespective of geography, with the inherent flexibility in aggregating estimates to different spatial units of interest, which makes it robust to changes in administrative boundaries. Also, because estimates can be produced at a high resolution (eg, 1 or 5 km), continuous GP models are well‐suited for estimating the heterogeneities that often exist in HDIs, which are typically masked when examining aggregate, administrative‐level estimates. Nevertheless, as we have demonstrated here, such high‐resolution estimates can have higher uncertainties, particularly in areas where cluster‐level data are sparse. As such, these estimates must be considered carefully when used for program planning in light of the associated uncertainties. An alternative approach to model‐based geostatistics, that is, the continuous GP modeling framework described here, is kriging interpolation.^46^ However, the approach is better suited to Gaussian data and there is evidence that it oversmooths the data and could, as a result, produce less reliable predictions.^47^, ^48^ Model‐based geostatistics allows the specification of an appropriate sampling distribution for the data, thus enabling better characterization of uncertainties in the data and uncertainties from other sources when implemented in a Bayesian framework. The continuous GP modeling framework is the state‐of‐the‐art method used by different research programs producing modeled estimates of HDIs using survey data, including the DHS program.^15^, ^16^, ^17^, ^19^, ^20^ Nevertheless, out of all the discrete and continuous spatial models investigated, we found that model D‐LN (ie, the logit‐normal model) performed best for district‐level estimation, corroborating findings elsewhere.^8^, ^22^

When producing estimates at the district level using the continuous GP models, we found that accounting for between‐cluster variation did not have any real effect on the estimates. Intuitively, this is to be expected since such micro‐scale variation is more likely to affect finer scale predictions (eg, the grid level) (see, eg, Dong and Wakefield^49^). When comparing predictive performance based on the use of weighted and unweighted data for model‐fitting, we found no major differences attributable to weighting among the discrete spatial models and between the discrete and continuous models. However, as stated previously, weighting is important in the discrete spatial models to guarantee the representativeness of the data. For the continuous GP models, it is thought that the use of population‐weighted aggregation when producing estimates at the administrative level helps to adjust for the uneven distribution of populations within the administrative units. While the use of geospatial covariates in continuous GP models to improve predictive performance has been a common practice,^15^, ^16^, ^17^, ^19^, ^20^, ^26^ in some settings where the interest is in comparisons between both classes of models, it can be difficult to find covariates that are informative across all the models, often due to the effect of spatial misalignment and/or the distribution of the outcome being modeled. This was particularly the case for DTP3/Malawi, where some covariates were significant predictors of coverage at the grid level but not at the district level (although the covariates yielded no meaningful improvement in predictive power due to the little variation in the data). Given that the estimated relationships between the covariates and vaccination coverage could also be confounded by aggregation bias in the models investigated here, caution must be observed when interpreting the estimated regression coefficients. Ideally, estimation of causal relationships between HDIs and covariate factors should be done using individual‐level data, possibly in a multilevel modeling framework.

Our analyses are subject to some limitations. First, limited sets of (geospatial) covariates were considered and utilized in our analyses. Often, broader sets of covariates are considered when producing maps of HDIs using continuous GP models.^15^, ^19^, ^20^ In particular, for MCV1/Kenya, the inclusion of more covariates in the analysis may result in additional improvements in the predictive ability of the continuous models. However, this is unlikely to be the case for DTP3/Malawi due to the little variation in the data. Secondly, there are other discrete spatial smoothing models not included in this study (see References 6, 8, 10), although these models are not without their own challenges, such as poorer performance in areas with small sample sizes and inability to produce estimates that are constrained to the unit interval.^6^ Thirdly, it is possible to investigate the predictive performance of both classes of models for district‐level estimation using data from a survey that is not representative at the district level. However, the challenge with such an approach is that the estimates that are produced can, perhaps, only be assessed using the associated uncertainties due to the lack of a gold standard or benchmark (the direct estimates are likely to be unreliable in this case) against which to compare these.

In conclusion, our study has shown that continuous GP models are a credible alternative to discrete spatial smoothing methods for producing district‐level estimates of vaccination coverage using household survey data.

## AUTHOR CONTRIBUTIONS

C. Edson Utazi conceptualized and designed the study. C. Edson Utazi, Kristine Nilsen, and Oliver Pannell contributed to data preparation and processing. C. Edson Utazi led the analysis and writing of the manuscript. All authors contributed to the initial draft of the manuscript. All authors contributed to writing and editing the manuscript.

## Supporting information


**AppendixS1** **: Supporting information**

## Data Availability

The DHS data analyzed in the work are available for download with permission from the DHS program (www.dhsprogram.com). The geospatial covariates and other data can be obtained from the sources provided. All code used in the analysis is available from https://figshare.com/articles/journal_contribution/Code_zip/7633538.
