# Supplementary material for: District‐level estimation of vaccination coverage: Discrete vs continuous spatial models
Source: Stat Med. 2021 Feb 4;40(9):2197–211. doi: 10.1002/sim.8897 (PMC8638675; doi:10.1002/sim.8897)
Supplement: Supplementary file 1 — AppendixS1 : Supporting information [file SIM-40-2197-s001.docx]

**District-level estimation of vaccination coverage: Discrete versus continuous spatial models**

C. Edson Utazi^a,b^, Kristine Nilsen^a^, Oliver Pannell^a^, Winfred Dotse-Gborgbortsi^a^, Andrew J. Tatem^a^

*^a^WorldPop, School of Geography and Environmental Science, University of Southampton, SO17 1BJ, UK*

*^b^Southampton Statistical Sciences Research Institute, University of Southampton, SO17 1BJ, UK*

**Supplementary information**

**Table 1: Description of covariates used in the analysis for MCV1/Kenya and DTP3/Malawi**

| Covariate | Description | Year | Source |
| --- | --- | --- | --- |
| Kenya | | | |
| Travel time to the nearest health facility | Travel time (in minutes) from each 5 km^2^ grid to the nearest health facility created using a health facility data base from Maina *et al*. (2019) and the methodology described in Weiss *et al*. (2018) | 2018 | Maina, J. *et al.* A spatial database of health facilities managed by the public health sector in sub Saharan Africa. *Sci Data* **6,**134 (2019). https://doi.org/10.1038/s41597-019-0142-2  Weiss, D.J. *et al*. (2018). A global map of travel time to cities to access inequalities in accessibility in 2015. *Nature* 553(7688):333‐336. doi:10.1038/nature25181. |
| Night-time lights | VIIRS night-time lights  Nano-watts (sqcm*sr) | 2016 | NOAA – Visible Infrared Imaging Radiometer Suite. https://ngdc.noaa.gov/eog/viirs/index.html |
| Poultry density | No. of chickens per 5 km^2^ grid | 2010 | Gilbert, M. *et al.*  (2018) Global Distribution Data for Cattle, Buffaloes, Horses, Sheep, Goats, Pigs, Chickens and Ducks in 2010. Nature Scientific data, 5:180227. doi: 10.1038/sdata.2018.227 |
| Slope | Slope (in degrees) | 2000 | De Ferranti, J. (2000). Digital elevation data (www.viewfinderpanoramas.org/dem3.html); based on NASA’s Shuttle Radar Topography Mission (SRTM) data (http://www2.jpl.nasa.gov/srtm/) |
| Malawi | | | |
| Ownership of health card/document | Proportion of children age <= 35 months who owned a vaccination card and/or a health document which were/was seen during the survey | 2015-16 | National Statistical Office, Malawi and ICF. 2017. Malawi Demographic and Health Survey 2015-16. Zomba, Malawi: National Statistical Office and ICF. Available at http://dhsprogram.com/pubs/pdf/FR319/FR319.pdf. |
| Household wealth | Proportion of households (with at least one living child) belonging to the top three wealth quintiles (middle/richer/richest) | 2015-16 | National Statistical Office, Malawi and ICF. 2017. Malawi Demographic and Health Survey 2015-16. Zomba, Malawi: National Statistical Office and ICF. Available at http://dhsprogram.com/pubs/pdf/FR319/FR319.pdf. |
| Mother’s education | Proportion of mothers who had at least a primary education | 2015-16 | National Statistical Office, Malawi and ICF. 2017. Malawi Demographic and Health Survey 2015-16. Zomba, Malawi: National Statistical Office and ICF. Available at http://dhsprogram.com/pubs/pdf/FR319/FR319.pdf. |
| Night-time lights | VIIRS night-time lights  Nano-watts (sqcm*sr) | 2016 | NOAA – Visible Infrared Imaging Radiometer Suite. https://ngdc.noaa.gov/eog/viirs/index.html |
| Travel time to cities of at least 50,000 people | Travel time in minutes | 2015 | Weiss, D.J. *et al*. (2018). A global map of travel time to cities to access inequalities in accessibility in 2015. *Nature*. |

^a^The DHS program creates wealth quintiles from standardized scores generated through principal component analysis involving variables on household assets; see https://dhsprogram.com/topics/wealth-index/.


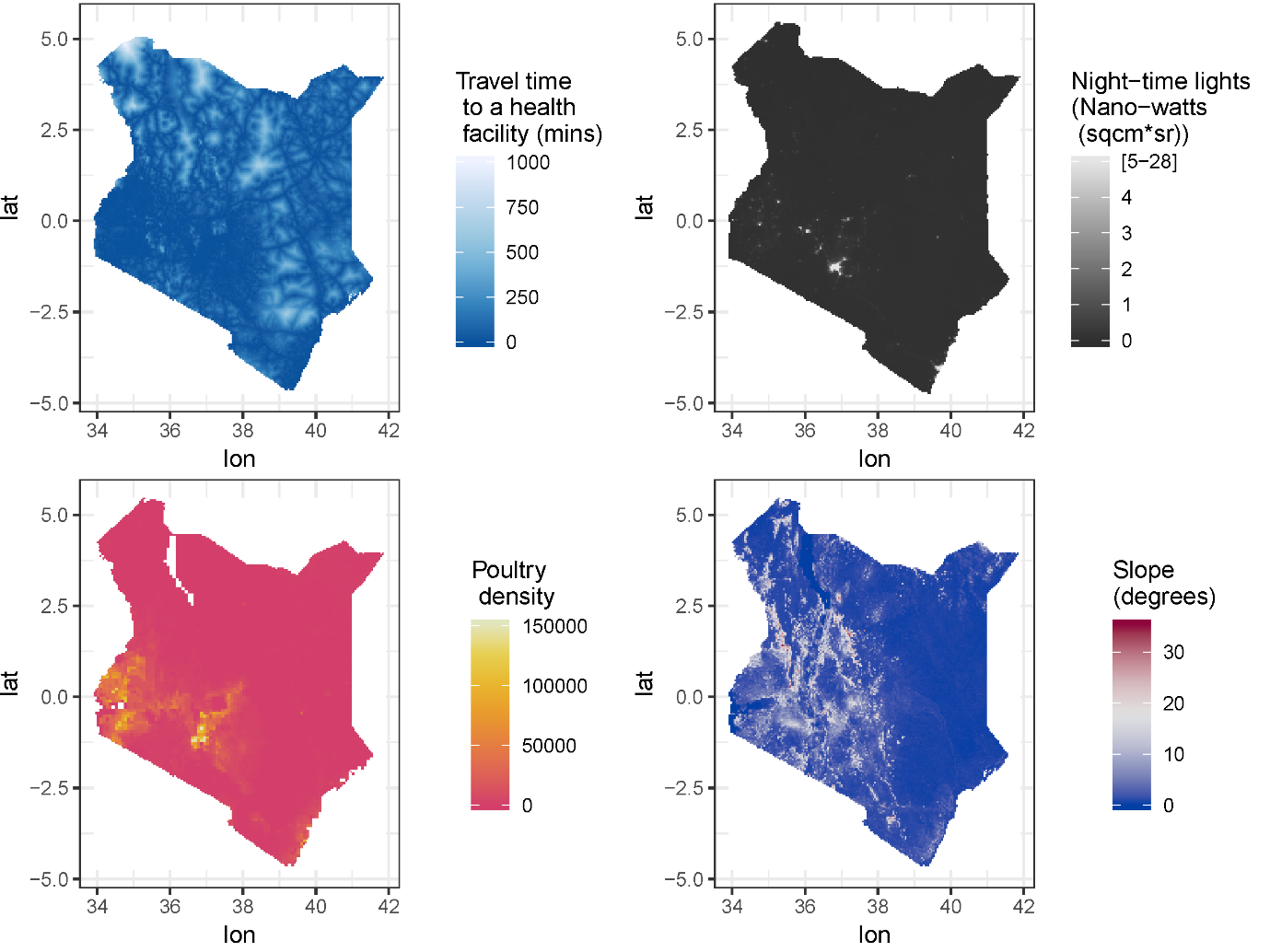


Figure 1: Plots of geospatial covariates used for model-fitting for MCV1/Kenya

**
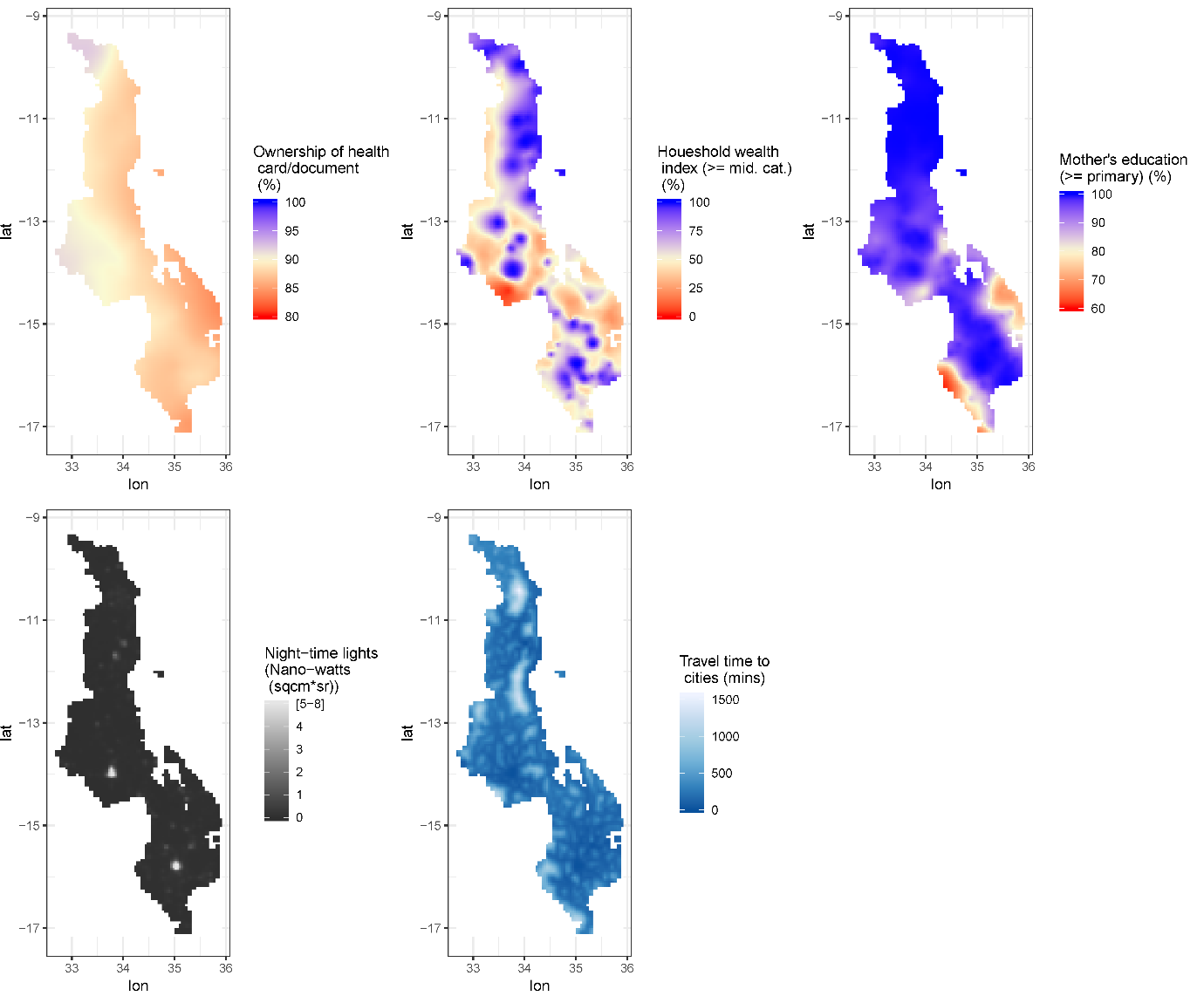
**

Figure 2: Plots of interpolated DHS and geospatial covariates used for model-fitting for DTP3/Malawi

**Table 2: Posterior estimates of parameters of the fitted models for MCV1/Kenya (Covariates included)**

| **Parameter estimates for model D-UNWB** | | | | | |
| --- | --- | --- | --- | --- | --- |
| Parameter | Mean | Std. Dev. | 2.5% | 50% | 97.5% |
| Intercept | 3.8708 | 1.8056 | 0.2929 | 3.8751 | 7.4205 |
| log(Poultry density) | -0.0320 | 0.1445 | -0.3154 | -0.0327 | 0.2551 |
| log(Slope) | 0.1799 | 0.1736 | -0.1575 | 0.1778 | 0.5294 |
| log(Night-time lights) | -0.1529 | 0.1206 | -0.3945 | -0.1517 | 0.0819 |
| log(Travel time) | -0.7678 | 0.2104 | -1.1845 | -0.7673 | -0.3542 |
| $\sigma_{\phi}^{2}$ | 0.6039 | 0.2393 | 0.2621 | 0.5613 | 1.1888 |
| $\rho$ | 0.7526 | 0.1713 | 0.3376 | 0.7915 | 0.9716 |
| **Parameter estimates for model D-LN** | | | | | |
| Parameter | Mean | Std. Dev. | 2.5% | 50% | 97.5% |
| Intercept | 3.1948 | 1.9013 | -0.5777 | 3.1999 | 6.9356 |
| log(Poultry density) | -0.0005 | 0.1529 | -0.3006 | -0.0013 | 0.3036 |
| log(Slope) | 0.1048 | 0.1840 | -0.2501 | 0.1015 | 0.4783 |
| log(Night-time lights) | -0.1126 | 0.1288 | -0.3716 | -0.1109 | 0.1372 |
| log(Travel time) | -0.6026 | 0.2270 | -1.0536 | -0.6018 | -0.1566 |
| $\sigma_{\phi}^{2}$ | 0.6032 | 0.2715 | 0.2295 | 0.5512 | 1.2764 |
| $\rho$ | 0.6550 | 0.2180 | 0.1831 | 0.6921 | 0.9614 |
| **Parameter estimates for model D-ESS** | | | | | |
| Parameter | Mean | Std. Dev. | 2.5% | 50% | 97.5% |
| Intercept | 3.6072 | 2.1597 | -0.6603 | 3.6074 | 7.8690 |
| log(Poultry density) | -0.0165 | 0.1738 | -0.3591 | -0.0168 | 0.3272 |
| log(Slope) | 0.1748 | 0.2098 | -0.2347 | 0.1728 | 0.5955 |
| log(Night-time lights) | -0.1605 | 0.1464 | -0.4523 | -0.1596 | 0.1260 |
| log(Travel time) | -0.7160 | 0.2567 | -1.2237 | -0.7158 | -0.2097 |
| $\sigma_{\phi}^{2}$ | 0.8710 | 0.3509 | 0.3731 | 0.8077 | 1.7316 |
| $\rho$ | 0.6325 | 0.2212 | 0.1681 | 0.6640 | 0.9558 |
| **Parameter estimates for model C-GPIID** | | | | | |
| Parameter | Mean | Std. Dev. | 2.5% | 50% | 97.5% |
| Intercept | 2.0595 | 0.3657 | 1.3290 | 2.0614 | 2.7758 |
| log(Poultry density) | 0.0423 | 0.0247 | -0.0073 | 0.0427 | 0.0898 |
| log(Slope) | 0.1344 | 0.0761 | -0.0144 | 0.1342 | 0.2845 |
| log(Night-time lights) | 0.0004 | 0.0841 | -0.1627 | -0.0003 | 0.1672 |
| log(Travel time) | -0.3439 | 0.0810 | -0.5025 | -0.3440 | -0.1847 |
| Range* | 2.2394 | 0.8355 | 1.0438 | 2.0894 | 4.2850 |
| Spatial variance | 0.4756 | 0.1777 | 0.2147 | 0.4463 | 0.9043 |
| iid variance | 0.1066 | 0.0722 | 0.0148 | 0.0912 | 0.2814 |
| **Parameter estimates for model C-GP** | | | | | |
| Parameter | Mean | Std. Dev. | 2.5% | 50% | 97.5% |
| Intercept | 2.0036 | 0.3612 | 1.2805 | 2.0070 | 2.7043 |
| log(Poultry density) | 0.0419 | 0.0239 | -0.0063 | 0.0422 | 0.0878 |
| log(Slope) | 0.1326 | 0.0736 | -0.0111 | 0.1323 | 0.2779 |
| log(Night-time lights) | 0.0090 | 0.0820 | -0.1498 | 0.0082 | 0.1719 |
| log(Travel time) | -0.3276 | 0.0774 | -0.4788 | -0.3279 | -0.1749 |
| Range* | 2.0460 | 0.7654 | 0.9962 | 1.8911 | 3.9599 |
| Spatial variance | 0.4869 | 0.1672 | 0.2330 | 0.4621 | 0.8830 |

*in decimal degrees

**Table 3: Posterior estimates of parameters of the fitted models for MCV1/Kenya (Covariates excluded)**

| **Parameter estimates for model D-UNWB** | | | | | |
| --- | --- | --- | --- | --- | --- |
| Parameter | Mean | Std. Dev. | 2.5% | 50% | 97.5% |
| Intercept | 2.0385 | 0.4948 | 1.0353 | 2.0355 | 3.0530 |
| $\sigma_{\phi}^{2}$ | 1.3531 | 0.4303 | 0.7061 | 1.2854 | 2.3809 |
| $\rho$ | 0.7444 | 0.1638 | 0.3573 | 0.7763 | 0.9657 |
| **Parameter estimates for model D-LN** | | | | | |
| Parameter | Mean | Std. Dev. | 2.5% | 50% | 97.5% |
| Intercept | 1.9294 | 0.4018 | 1.1207 | 1.9248 | 2.7577 |
| $\sigma_{\phi}^{2}$ | 1.0747 | 0.3923 | 0.5043 | 1.0075 | 2.0271 |
| $\rho$ | 0.6911 | 0.1896 | 0.2665 | 0.7228 | 0.9592 |
| **Parameter estimates for model D-ESS** | | | | | |
| Parameter | Mean | Std. Dev. | 2.5% | 50% | 97.5% |
| Intercept | 2.0385 | 0.4948 | 1.0353 | 2.0355 | 3.0530 |
| $\sigma_{\phi}^{2}$ | 1.3531 | 0.4303 | 0.7061 | 1.2854 | 2.3809 |
| $\rho$ | 0.7444 | 0.1638 | 0.3573 | 0.7763 | 0.9657 |
| **Parameter estimates for model C-GPIID** | | | | | |
| Parameter | Mean | Std. Dev. | 2.5% | 50% | 97.5% |
| Intercept | 1.3453 | 0.2712 | 0.7724 | 1.3548 | 1.8631 |
| Range* | 1.8284 | 0.4869 | 1.0722 | 1.7586 | 2.9724 |
| Spatial variance | 0.9276 | 0.2692 | 0.5212 | 0.8851 | 1.5702 |
| iid variance | 0.1038 | 0.0793 | 0.0108 | 0.0838 | 0.3016 |
| **Parameter estimates for model C-GP** | | | | | |
| Parameter | Mean | Std. Dev. | 2.5% | 50% | 97.5% |
| Intercept | 1.3228 | 0.2635 | 0.7675 | 1.3328 | 1.8191 |
| Range* | 1.5956 | 0.4057 | 0.9827 | 1.5302 | 2.5637 |
| Spatial variance | 0.9330 | 0.2405 | 0.5487 | 0.9028 | 1.4877 |

*in decimal degrees

**Table 4: Posterior estimates of parameters of the fitted models for DTP3/Malawi (covariates included)**

| **Parameter estimates for model D-UNWB** | | | | | |
| --- | --- | --- | --- | --- | --- |
| Parameter | Mean | Std. Dev. | 2.5% | 50% | 97.5% |
| Intercept | -0.7476 | 3.5776 | -7.7640 | -0.7682 | 6.3872 |
| Ownership of health card and/or document | 6.3999 | 3.5607 | -0.6469 | 6.4022 | 13.4199 |
| Household wealth | -1.1960 | 1.4137 | -4.1132 | -1.1588 | 1.5041 |
| log(Mother’s education) | 1.8353 | 1.3457 | -0.8466 | 1.8412 | 4.4832 |
| log(Night-time lights) | 0.0278 | 0.2374 | -0.4357 | 0.0254 | 0.5042 |
| log(Travel time) | -0.1091 | 0.4666 | -1.0274 | -0.1112 | 0.8194 |
| $\sigma_{\phi}^{2}$ | 0.3505 | 0.2493 | 0.0697 | 0.2870 | 1.0021 |
| $\rho$ | 0.5160 | 0.2548 | 0.0705 | 0.5215 | 0.9399 |
| **Parameter estimates for model D-LN** | | | | | |
| Parameter | Mean | Std. Dev. | 2.5% | 50% | 97.5% |
| Intercept | 0.1650 | 3.6561 | -7.0282 | 0.1501 | 7.4357 |
| Ownership of health card and/or document | 4.8639 | 3.6186 | -2.2798 | 4.8602 | 12.0184 |
| Household wealth | -1.5929 | 1.5225 | -4.6967 | -1.5622 | 1.3350 |
| log(Mother’s education) | 1.9994 | 1.4119 | -0.8146 | 2.0060 | 4.7726 |
| log(Night-time lights) | 0.0110 | 0.2597 | -0.5010 | 0.0106 | 0.5241 |
| log(Travel time) | -0.0254 | 0.5067 | -1.0287 | -0.0248 | 0.9730 |
| $\sigma_{\phi}^{2}$ | 0.3414 | 0.2554 | 0.0626 | 0.2742 | 1.0137 |
| $\rho$ | 0.5143 | 0.2525 | 0.0723 | 0.5192 | 0.9370 |
| **Parameter estimates for model D-ESS** | | | | | |
| Parameter | Mean | Std. Dev. | 2.5% | 50% | 97.5% |
| Intercept | 0.4092 | 3.8410 | -7.1165 | 0.3830 | 8.0855 |
| Ownership of health card and/or document | 4.7080 | 3.8288 | -2.8531 | 4.7031 | 12.2814 |
| Household wealth | -1.8748 | 1.5174 | -5.0604 | -1.8158 | 0.9683 |
| log(Mother’s education) | 2.2122 | 1.4632 | -0.6983 | 2.2157 | 5.1010 |
| log(Night-time lights) | 0.0098 | 0.2589 | -0.4907 | 0.0056 | 0.5346 |
| log(Travel time) | -0.0010 | 0.5109 | -1.0079 | -0.0033 | 1.0167 |
| $\sigma_{\phi}^{2}$ | 0.4431 | 0.2984 | 0.0972 | 0.3690 | 1.2223 |
| $\rho$ | 0.5252 | 0.2511 | 0.0781 | 0.5336 | 0.9397 |
| **Parameter estimates for model C-GPIID** | | | | | |
| Parameter | Mean | Std. Dev. | 2.5% | 50% | 97.5% |
| Intercept | 0.9456 | 0.7822 | -0.5865 | 0.9435 | 2.4872 |
| Ownership of health card and/or document | 3.1589 | 0.5290 | 2.1222 | 3.1581 | 4.1989 |
| Household wealth | -0.7171 | 0.4160 | -1.5354 | -0.7167 | 0.0981 |
| log(Mother’s education) | 1.2319 | 0.3861 | 0.4642 | 1.2350 | 1.9817 |
| log(Night-time lights) | 0.1259 | 0.1057 | -0.0790 | 0.1249 | 0.3360 |
| log(Travel time) | 0.0537 | 0.1361 | -0.2129 | 0.0533 | 0.3217 |
| Range* | 0.6411 | 0.3065 | 0.2493 | 0.5718 | 1.4275 |
| Spatial variance | 0.2044 | 0.1481 | 0.0412 | 0.1653 | 0.5972 |
| iid variance | 0.1532 | 0.1110 | 0.0190 | 0.1264 | 0.4283 |
| **Parameter estimates for model C-GP** | | | | | |
| Parameter | Mean | Std. Dev. | 2.5% | 50% | 97.5% |
| Intercept | 0.8767 | 0.7586 | -0.6103 | 0.8749 | 2.3728 |
| Ownership of health card and/or document | 3.1291 | 0.5034 | 2.1408 | 3.1290 | 4.1170 |
| Household wealth | -0.7214 | 0.3994 | -1.5065 | -0.7212 | 0.0619 |
| log(Mother’s education) | 1.2102 | 0.3624 | 0.4856 | 1.2146 | 1.9103 |
| log(Night-time lights) | 0.1245 | 0.1015 | -0.0718 | 0.1235 | 0.3268 |
| log(Travel time) | 0.0523 | 0.1310 | -0.2034 | 0.0516 | 0.3119 |
| Range* | 0.5801 | 0.2604 | 0.2550 | 0.5178 | 1.2544 |
| Spatial variance | 0.2395 | 0.1366 | 0.0620 | 0.2110 | 0.5831 |

*in decimal degrees

**Table 5:** **Posterior estimates of parameters of the fitted models for DTP3/Malawi (covariates excluded)**

| **Parameter estimates for model D-UNWB** | | | | | |
| --- | --- | --- | --- | --- | --- |
| Parameter | Mean | Std. Dev. | 2.5% | 50% | 97.5% |
| Intercept | 2.7411 | 0.2402 | 2.2714 | 2.7363 | 3.2344 |
| $\sigma_{\phi}^{2}$ | 0.4141 | 0.2428 | 0.1145 | 0.3583 | 1.0378 |
| $\rho$ | 0.4550 | 0.2439 | 0.0616 | 0.4401 | 0.9071 |
| **Parameter estimates for model D-LN** | | | | | |
| Parameter | Mean | Std. Dev. | 2.5% | 50% | 97.5% |
| Intercept | 2.6412 | 0.2318 | 2.1917 | 2.6364 | 3.1142 |
| $\sigma_{\phi}^{2}$ | 0.3721 | 0.2340 | 0.0915 | 0.3162 | 0.9787 |
| $\rho$ | 0.4545 | 0.2484 | 0.0564 | 0.4390 | 0.9121 |
| **Parameter estimates for model D-ESS** | | | | | |
| Parameter | Mean | Std. Dev. | 2.5% | 50% | 97.5% |
| Intercept | 2.7443 | 0.2460 | 2.2605 | 2.7405 | 3.2461 |
| $\sigma_{\phi}^{2}$ | 0.4505 | 0.2662 | 0.1226 | 0.3892 | 1.1342 |
| $\rho$ | 0.4408 | 0.2453 | 0.0552 | 0.4211 | 0.9038 |
| **Parameter estimates for model C-GPIID** | | | | | |
| Parameter | Mean | Std. Dev. | 2.5% | 50% | 97.5% |
| Intercept | 2.7991 | 0.1731 | 2.4467 | 2.7994 | 3.1394 |
| Range* | 0.9164 | 0.5714 | 0.2901 | 0.7603 | 2.4443 |
| Spatial variance | 0.2206 | 0.1448 | 0.0455 | 0.1868 | 0.5934 |
| iid variance | 0.3970 | 0.1924 | 0.1419 | 0.3571 | 0.8821 |
| **Parameter estimates for model C-GP** | | | | | |
| Parameter | Mean | Std. Dev. | 2.5% | 50% | 97.5% |
| Intercept | 2.6577 | 0.1812 | 2.2848 | 2.6622 | 2.9900 |
| Range* | 0.5813 | 0.2571 | 0.2537 | 0.5219 | 1.2424 |
| Spatial variance | 0.3309 | 0.1507 | 0.1168 | 0.3048 | 0.6975 |

*in decimal degrees


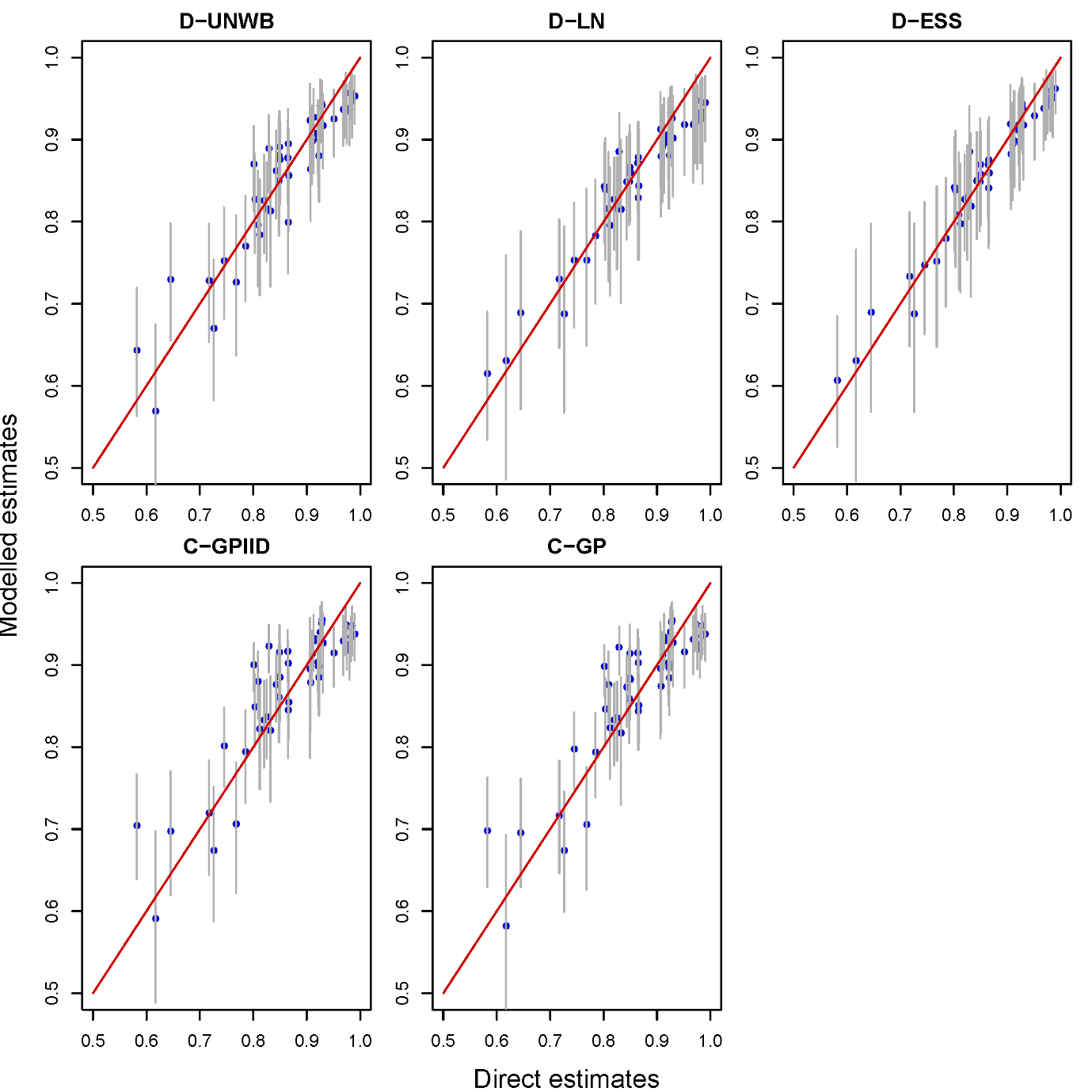


Figure 3: Plots of district level direct estimates versus in-sample modelled estimates of MCV1 coverage (Kenya) for children aged 12-23 months produced using the various models investigated.


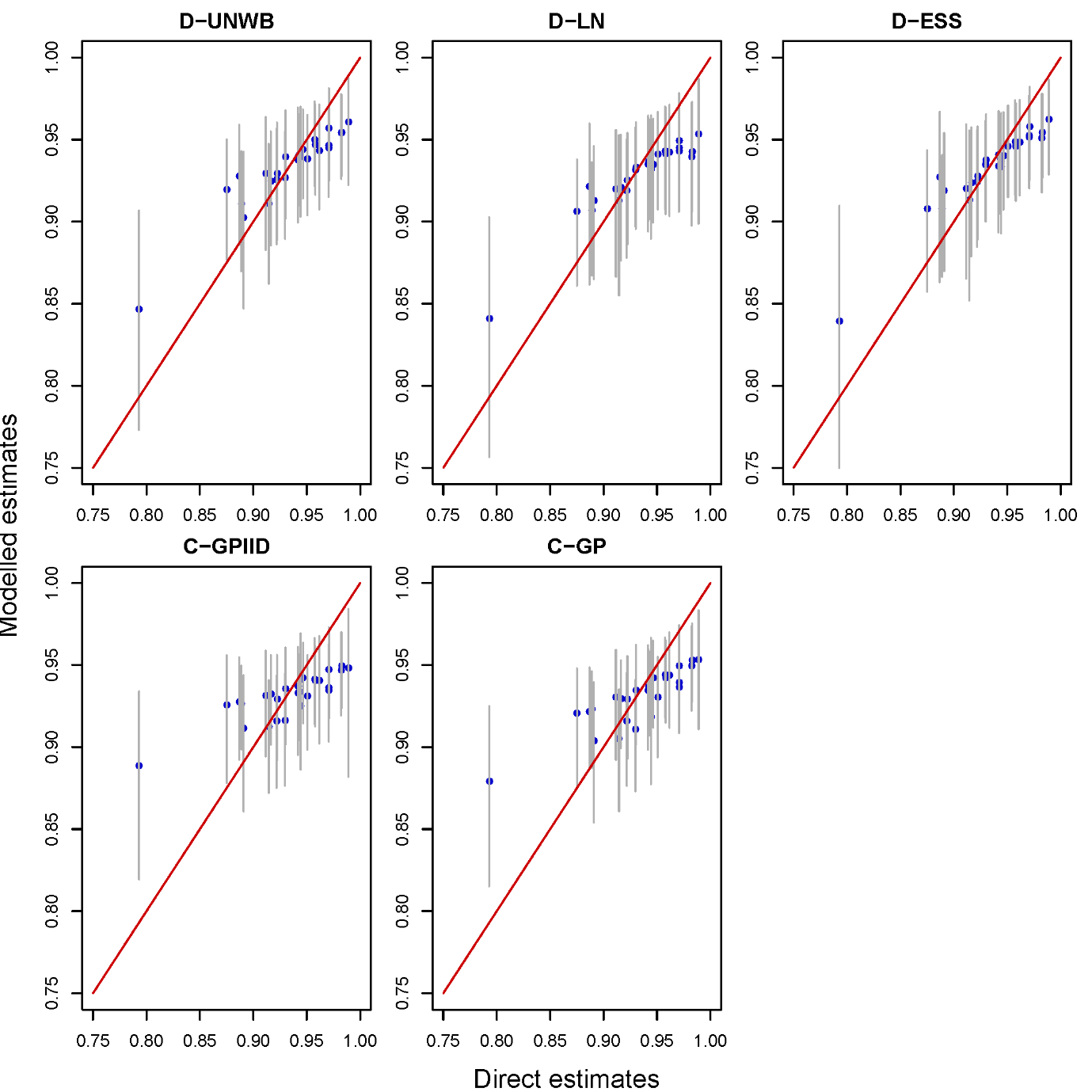


Figure 4: Plots of district level direct estimates versus in-sample modelled estimates of DTP3 coverage (Malawi) for children aged 12-23 months produced using the various models investigated.


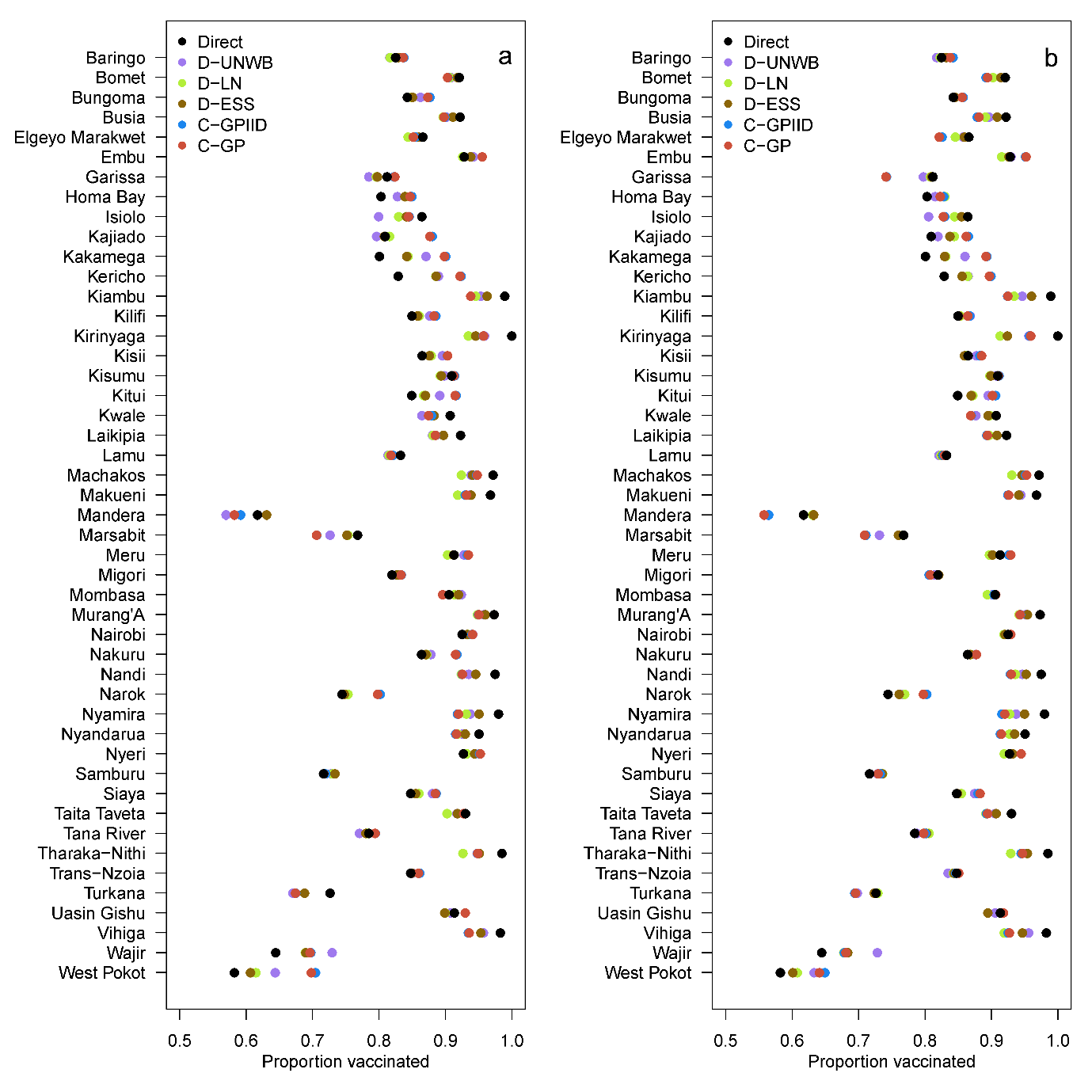


Figure 5: Plots of estimates of MCV1 coverage (Kenya) for children aged 12-23 months produced using the different approaches investigated (a) with covariates and (b) without covariates included in the analysis. The direct survey estimates are the same in (a) and (b).


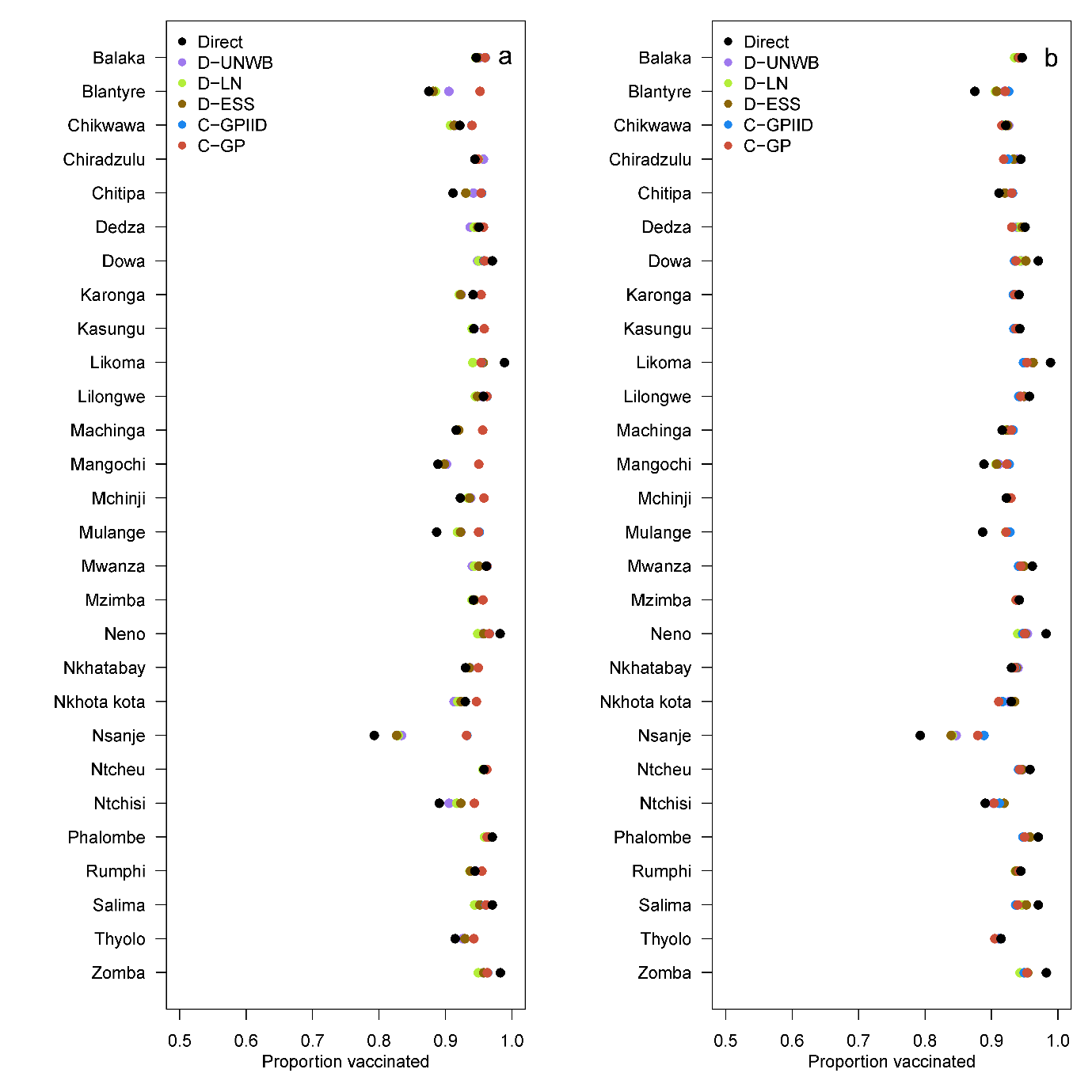


Figure 6: Plots of estimates of DTP3 coverage (Malawi) for children aged 12-23 months produced using the different approaches investigated (a) with covariates and (b) without covariates included in the analysis. The direct survey estimates are the same in (a) and (b).


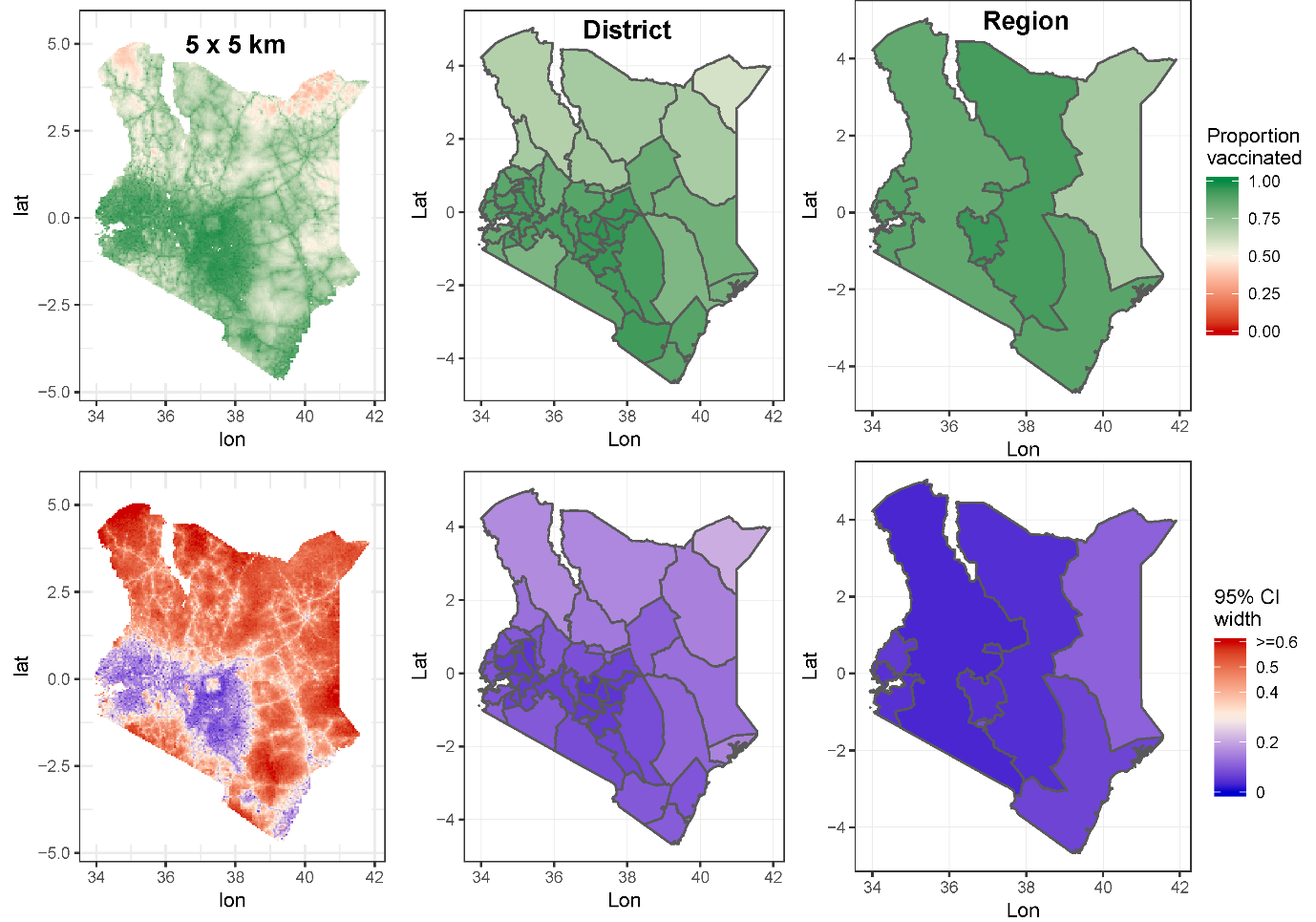


Figure 7: (Top panels) Maps of 5 x 5 km estimates of MCV1 coverage (Kenya) among children aged 12-23 months produced using model C-GPIID and corresponding aggregated district and provincial/regional estimates. The corresponding uncertainties are shown as the widths of the 95% credible intervals of the estimates (bottom panels).


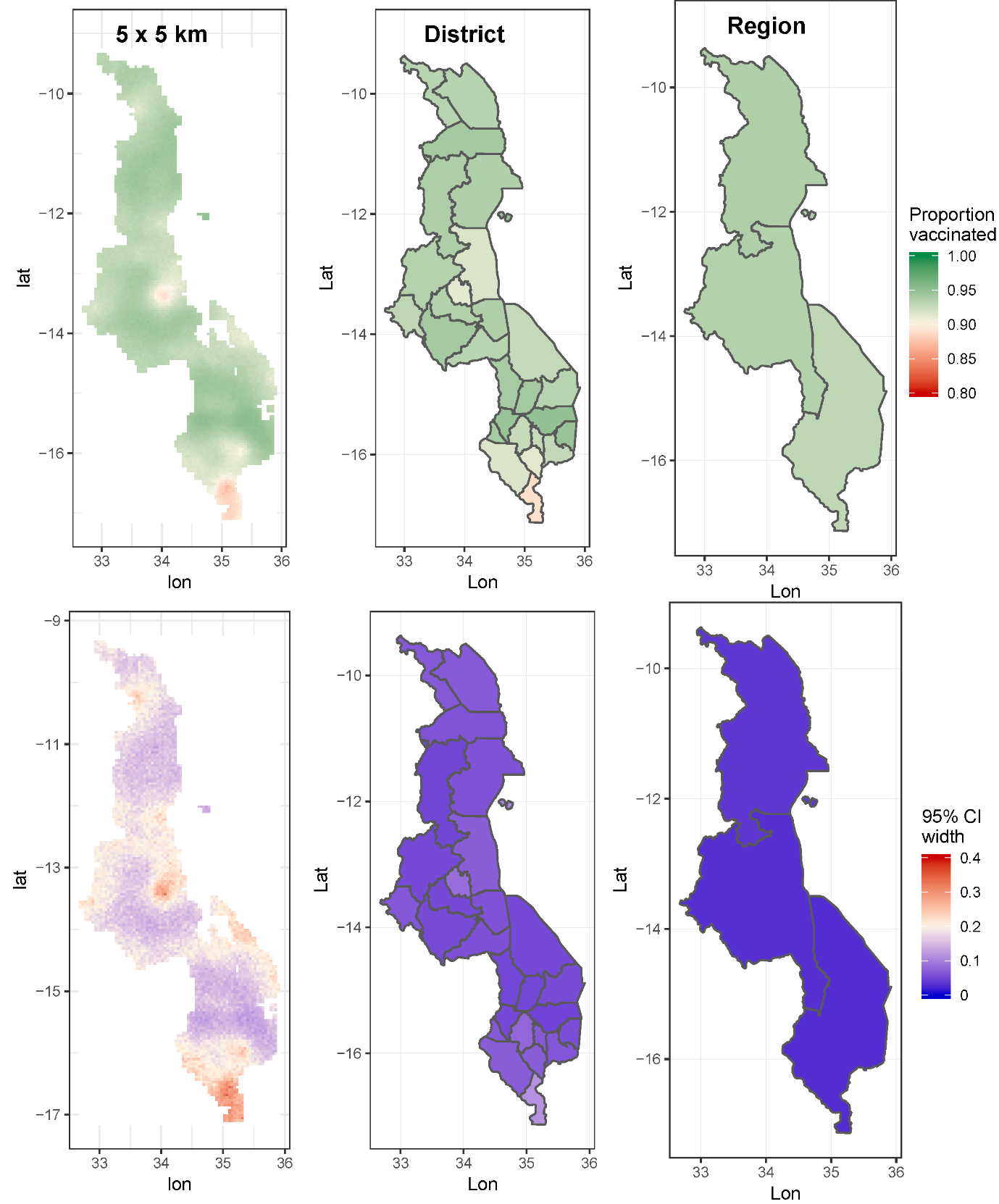


Figure 8: (Top panels) Maps of 5 x 5 km estimates of DTP3 coverage (Malawi) among children aged 12-23 months produced using model C-GPIID and corresponding aggregated district and provincial/regional estimates. The corresponding uncertainties are shown as the widths of the 95% credible intervals of the estimates (bottom panels).

**Additional information on the INLA-SPDE approach and model fitting**

The SPDE approach involves a triangulation of the spatial domain in order to approximate $\boldsymbol{\omega.}$ A mesh was constructed for this approximation using the survey cluster locations and the boundary points of Kenya and Malawi. For Kenya, the maximum triangle edge length was set to be 0.2 degrees in the inner mesh (which is smaller than $r_{0}\approx0.51 \mathrm{degrees}$) and 0.4 degrees in the outer mesh. For Malawi, the edge lengths were 0.07 degrees in the inner mesh (which is smaller than $r_{0}\approx0.40 \mathrm{degrees}$) and 0.3 degrees in the outer mesh. The choice of these edge lengths was guided by the need to maintain a balance between the accuracy of the approximation and computational costs.

From each of the fitted models and country/vaccine, we generated 1000 samples from the posterior distributions of the parameters of the model, as well as from the posterior predictive distributions of vaccination coverage for each of the prediction locations, i.e. the 5 x 5 km grid cells for models C-GPIID and C-GP and the districts for models –D-UNWB, D-LN and D-ESS. In the continuous GP models, these samples were then summarized to produce the grid level estimates and aggregated to produce estimates at the district and regional levels as described in the manuscript. For the district models, the posterior samples were only required to calculate probabilities of reaching specified coverage thresholds in each district, as other model outputs are produced by default in R-INLA.


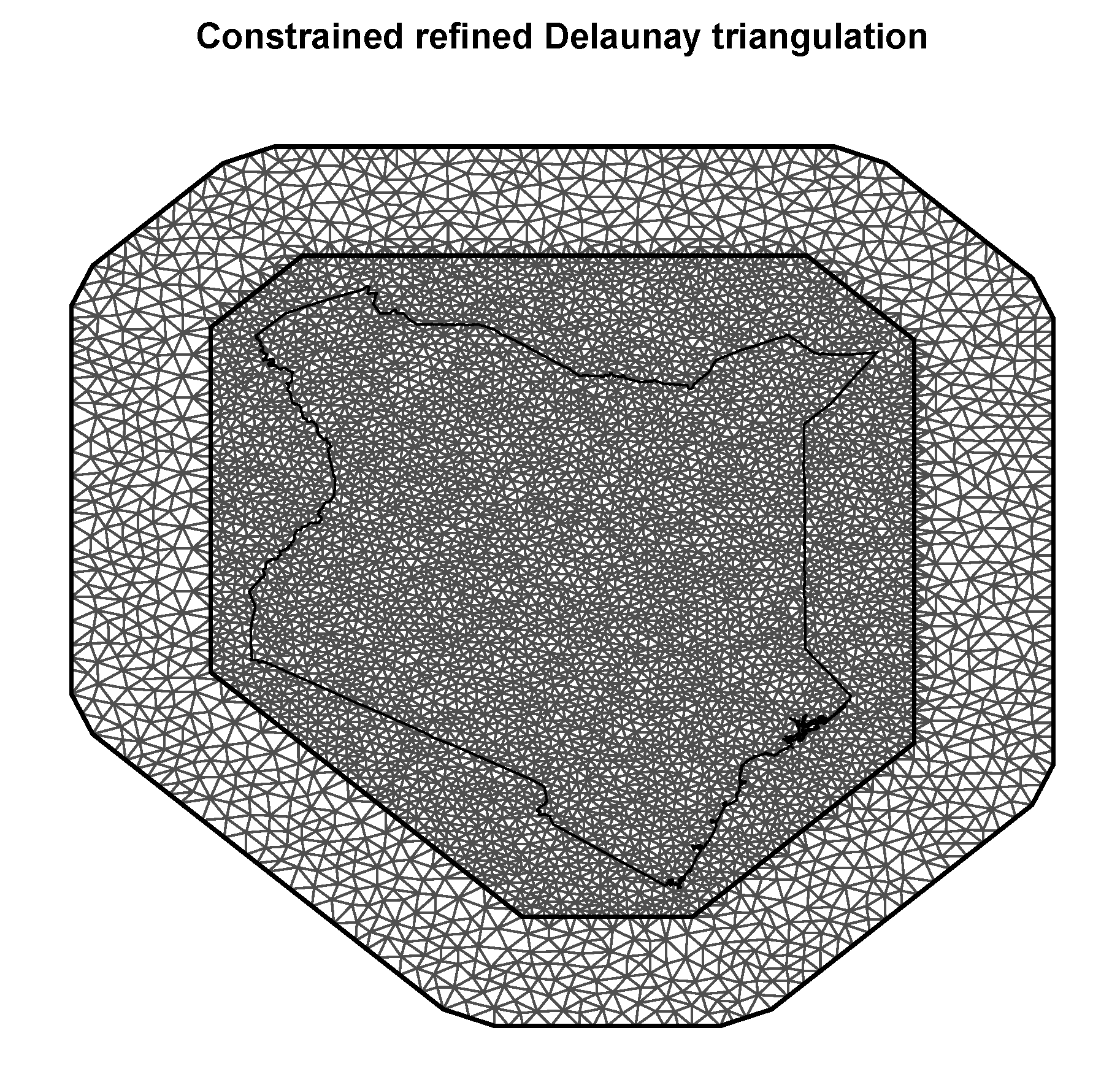


Figure 5: An Example fine triangulation mesh for models C-GPIID and C-GP for MCV1/Kenya.
